# Maternal low and high hemoglobin concentrations and associations with adverse maternal and infant health outcomes: an updated global systematic review and meta-analysis

**DOI:** 10.1186/s12884-023-05489-6

**Published:** 2023-04-19

**Authors:** Melissa F. Young, Brietta M. Oaks, Hannah Paige Rogers, Sonia Tandon, Reynaldo Martorell, Kathryn G. Dewey, Amanda S. Wendt

**Affiliations:** 1 Hubert Department of Global Health, Emory University, 1518 Clifton Road NE, 30322 Atlanta, GA USA; 2 Department of Nutrition and Food Sciences, University of Rhode Island, 02881 Kingston, United States; 3 Department of Nutrition, University of California, Davis, 95616 Davis, United States; 4 Research Department 2, Potsdam Institute for Climate Impact Research (PIK), Member of the Leibniz Association, PO Box 60 12 03, 14412, Potsdam, Germany

**Keywords:** Hemoglobin, anemia, Pregnancy, Review, Birth outcomes

## Abstract

**Background:**

Growing evidence suggests low and high maternal hemoglobin (Hb) concentrations may have adverse consequences for maternal and child health. There remain questions on specific Hb thresholds to define anemia and high Hb as well as how cutoffs may vary by anemia etiology and timing of assessment.

**Methods:**

We conducted an updated systematic review (using PubMed and Cochrane Review) on low (< 110 g/L) and high (≥ 130 g/L) maternal Hb concentrations and associations with a range of maternal and infant health outcomes. We examined associations by timing of Hb assessment (preconception; first, second, and third trimesters, as well as at any time point in pregnancy), varying cutoffs used for defining low and high hemoglobin concentrations and performed stratified analyses by iron-deficiency anemia. We conducted meta-analyses to obtain odds ratios (OR) and 95% confidence intervals.

**Results:**

The updated systematic review included 148 studies. Low maternal Hb at any time point in pregnancy was associated with: low birthweight, LBW (OR (95% CI) 1.28 (1.22–1.35)), very low birthweight, VLBW (2.15 (1.47–3.13)), preterm birth, PTB (1.35 (1.29–1.42)), small-for-gestational age, SGA (1.11 (1.02–1.19)), stillbirth 1.43 (1.24–1.65)), perinatal mortality (1.75 (1.28–2.39)), neonatal mortality (1.25 (1.16–1.34), postpartum hemorrhage (1.69 (1.45–1.97)), transfusion (3.68 (2.58–5.26)), pre-eclampsia (1.57 (1.23–2.01)), and prenatal depression (1.44 (1.24–1.68)). For maternal mortality, the OR was higher for Hb < 90 (4.83 (2.17–10.74)) than for Hb < 100 (2.87 (1.08–7.67)). High maternal Hb was associated with: VLBW (1.35 (1.16–1.57)), PTB (1.12 (1.00-1.25)), SGA (1.17 (1.09–1.25)), stillbirth (1.32 (1.09–1.60)), maternal mortality (2.01 (1.12–3.61)), gestational diabetes (1.71 (1.19–2.46)), and pre-eclampsia (1.34 (1.16–1.56)). Stronger associations were noted earlier in pregnancy for low Hb and adverse birth outcomes while the role of timing of high Hb was inconsistent. Lower Hb cutoffs were associated with greater odds of poor outcomes; for high Hb, data were too limited to identify patterns. Information on anemia etiology was limited; relationships did not vary by iron-deficiency anemia.

**Conclusion:**

Both low and high maternal Hb concentrations during pregnancy are strong predictors of adverse maternal and infant health outcomes. Additional research is needed to establish healthy reference ranges and design effective interventions to optimize maternal Hb during pregnancy.

**Supplementary Information:**

The online version contains supplementary material available at 10.1186/s12884-023-05489-6.

## Background

Anemia remains a persistent global health problem, impacting over 269 million children, 571 million women of reproductive age and 32 million pregnant women [1]. While iron deficiency is considered one of the leading causes of anemia, the etiology of anemia, including the role of other micronutrient deficiencies, infections, inflammation or hemoglobinopathies, may vary widely by setting [1]. Anemia has adverse health and developmental outcomes across the lifespan including increased risk of hemorrhaging during birth; delayed child growth, cognition, and motor development; reduced physical work capacity in adults; and elevated morbidity and mortality among older populations [2]. Given the significance of the public health problem, there have been global calls to reduce anemia, particularly among the most vulnerable and high-risk population groups [3]. In 2020, the prevalence of anemia among pregnant and nonpregnant women was included as a key indicator for the UN Sustainable Development Goals (SDGs) [4]. The World Health Assembly called for a 50% reduction in anemia prevalence among women of reproductive age (15–49 years) by 2025, although this may be extended to 2030 [3, 5]. However, there has been insufficient progress and the prevalence of anemia has remained high among women of reproductive age (31% in 2000 to 30% in 2019) and pregnant women (41% in 2000 to 36% in 2019) [1]. Much of the progress has been in reducing severe and moderate anemia, with limited change in the prevalence of mild anemia.

A critical component of tracking progress towards global targets is having an agreed upon definition of anemia by the global community. WHO Hb cutoffs for defining anemia are currently being re-examined [6]. The WHO anemia cutoffs are based on limited data, in particular for pregnant women, and lack global representativeness and are widely acknowledged as outdated as they do not account for gestational age-specific changes in plasma volume expansion. Current overall anemia cutoffs are defined as < 110 g/L during pregnancy, [7] or trimester-specific anemia cutoffs (first trimester: < 110 g/L; second trimester: < 105 g/L; third trimester: < 110 g/L) [8, 9]. Across the literature, there is often inconsistency in the range of Hb cutoffs used to define low and high Hb thresholds, which impacts the prevalence of the problem and likelihood of detecting relationships with adverse maternal and child outcomes [10]. Studies using multiple Hb cutoffs have reported that in some cases only lower cutoff values were associated with adverse outcomes [11–14]. Few studies have evaluated the long-term health impact of maternal Hb concentrations [15]. An exception is a recent analysis of data from the Fetal Growth Longitudinal Study of the INTERGROWTH-21st Project, a study of prospective, population-based data from Brazil, China, India, Italy, Kenya, Oman, the United Kingdom and the United States [16]. This analysis provides maternal Hb normative centiles during pregnancy associated with positive birth outcomes as well as adequate child growth and development across the first 1000 days. The results suggest Hb cutoffs during pregnancy that are lower than currently recommended by the WHO [16]. However, key knowledge gaps remain on the optimal thresholds of maternal Hb concentration related to both maternal and infant health, and whether those thresholds vary by anemia etiology or timing of measurement. In response to the WHO call to re-evaluate the evidence on hemoglobin thresholds to define anemia, we previously conducted a systematic review on maternal Hb concentrations and maternal and infant health outcomes including studies through October 2018 [15]. Since then, significant new research has been reported on outcomes of interest, such as maternal depression and maternal mortality, that were not included in the previous meta-analysis due to a paucity of studies.

The objective of this study was to conduct an updated systematic review and meta-analysis to examine the associations of low and high maternal Hb concentrations during pregnancy with adverse maternal and infant health outcomes. Analyses were stratified by timing of Hb assessment (preconception, first, second and third trimesters), Hb cutoff category, and etiology of anemia (iron-deficiency anemia/non-iron deficiency anemia).

## Methods

### Search Strategy

Our search criteria included studies of associations between maternal anemia or Hb concentrations, measured during pregnancy or preconception, and adverse maternal and infant health outcomes, as previously defined [15]. Maternal outcomes included blood transfusion, gestational diabetes, maternal mortality, postpartum hemorrhage, prenatal depression, postpartum depression, and preeclampsia. Infant outcomes included low birth weight (LBW; <2500 g), very low birth weight (VLBW; <1500 g), neonatal mortality, perinatal mortality, preterm birth (PTB; <37 weeks), small-for-gestational age (SGA), and stillbirth. Cochrane Review and PubMed were searched with no restrictions for study population, language, or date, building directly from our prior systematic review [15]. In addition, references from prior reviews were also reviewed to identify additional studies. To be eligible for inclusion, studies had to report associations between maternal Hb concentrations or anemia (defined by Hb cutoff) assessed during preconception or pregnancy, and at least one of the adverse outcomes. Only peer-reviewed studies adjusting for one or more confounders were included. Publication dates for the studies in the meta-analysis (including both prior review [15] and updated search combined) ranged from January 1990 to April 2021 and included a total sample size of 13,839,327 women across 148 studies.

### Data extraction and quality assessment

Reviews for title and abstract screening, full text review, and data extraction were conducted using Covidence systematic review software by two team members. Conflicts were resolved by a third team member (for title and abstract review) or team committee (for full text). Key information extracted from manuscript included study design, year, gestational age/trimester at time of assessment, Hb concentration cutoffs, adverse outcome measures and adjusted measures of association with 95% confidence intervals. To ensure data quality, 10% of data extractions were conducted in duplicate. Authors were contacted for missing information when possible.

### Data management and analyses

Data were stratified by low Hb (< 110 g/L) and high Hb (≥ 130 g/L) and by timing of Hb measurement: preconception, first (≤ 13 weeks), second (14–26 weeks), and third (≥ 27 weeks) trimesters. The overall pregnancy estimates for low (< 110 g/L) and high Hb (≥ 130 g/L) included Hb concentrations measured at any time point during pregnancy (including studies with and without gestational age data). The Hb cutoffs used to define anemia varied across studies; thus, we created standard cutoffs to conduct various analyses (≤ 70, ≤ 80, ≤90, ≤ 100, ≤110, ≥ 130, ≥140, ≥ 150, and ≥ 160 g/L) to create summary estimates across Hb concentrations. Each category was cumulative whereby the estimates reported for the ≥ 130 g/L category include measurements for all studies using an Hb cutoff of ≥ 130, including all studies in the ≥ 140, ≥150, and ≥ 160 g/L categories. The reference group varied across studies and was either a cutoff (e.g. ≥110 g/L) or reference range (e.g. 110–119 g/L). Separate meta-analyses were conducted for iron deficiency anemia (IDA) and non-IDA for LBW, PTB, and SGA. All analyses were conducted using STATA version 14.2 (Stata Corp. 2015, College Station, TX). Values reported as odds ratio (OR) and 95% confidence intervals with significance indicated at P < 0.05. Q- and I^2^ values over 50% indicated heterogeneity and a Q-statistic P-value of > 0.10 indicated substantial heterogeneity. Given the significant heterogeneity, we used random effect models and the inverse-variance method for weighting for the meta-analyses.

## Results

The systematic review identified 9874 studies, of which 57 duplicates were removed (See Supplementary Fig. 1, Additional File 1). After screening abstracts for eligibility, we conducted full text reviews on 1142 studies and excluded 994 based on inclusion and exclusion criteria (n = 743), inability to contact the study author (n = 38), unable to translate (n = 9), inability to retrieve the full text document (n = 26), study result duplication (n = 1), inclusion of other statistical measures incompatible for meta-analysis (n = 61), other outcomes not included in review or insufficient for meta-analysis (n = 90) or conducted among high risk populations (n = 26). In total, we included 148 studies in the meta-analysis (See Supplementary Fig. 1, Additional File 1) [11, 17–163].

### Study characteristics

**Fig. 1:**
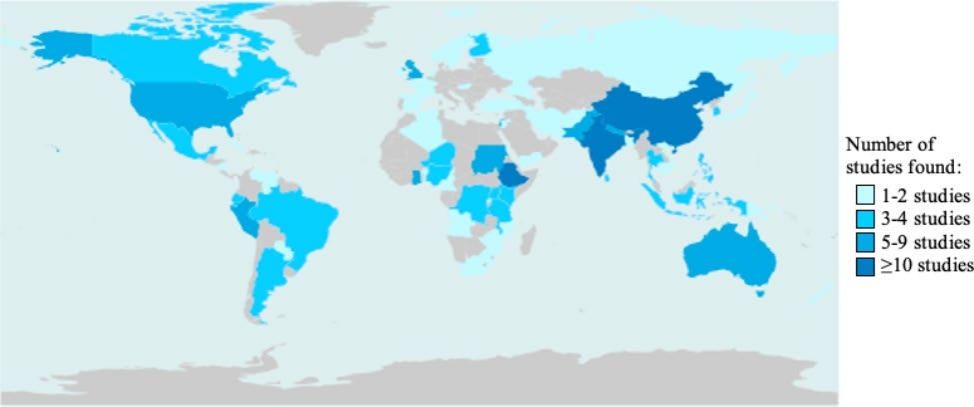
Global distribution of sites used in included studies (Analysis of the data was done using the rworldmap package v1.3-6 (1): https://www.rdocumentation.org/packages/rworldmap/versions/1.3-6 )

Among eligible studies, 43 were prospective cohorts, 50 were retrospective cohorts, 36 were case-control, and 19 were cross-sectional studies. This included an additional 53 new studies, including data from 32 new countries from the prior review [15]. As illustrated in Fig. 1 our combined updated review includes data from a total of 69 countries. Across the included countries there was variation in data available with China, Ethiopia, and India having over 10 studies and countries such as Afghanistan, Mozambique, and Nicaragua having only 1–2 studies and many countries none. The sample sizes from included studies ranged from 124 to 2,869,415 and the total sample size across all studies was 13,839,327 women. There was insufficient data to conduct meta-analyses on long-term outcomes. Limited data was available on preconception Hb concentrations and birth outcomes.

### Infant health outcomes

Birth outcomes with sufficient data for meta-analysis included: (1) LBW (n = 61); (2) PTB (n = 63); (3) SGA (n = 37); (4) stillbirth (n = 27); (5) perinatal mortality (n = 13); and (6) neonatal mortality (n = 10).

**Fig. 2:**
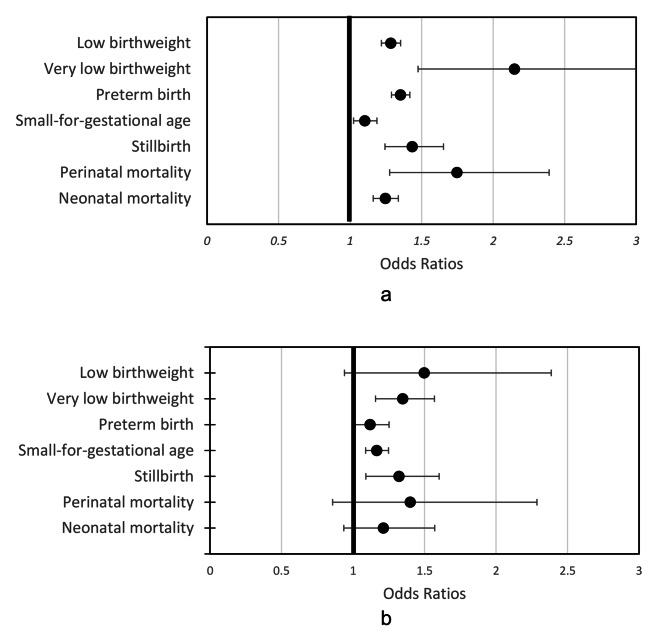
Summary estimates of low (**a**) and high (**b**) hemoglobin concentrations and associations with adverse infant outcomes

Figure 2 provides an overview of associations between maternal Hb and infant health outcomes, which are further described in Table 1 and Table 2 for low and high maternal Hb and stratified by Hb cutoff and timing.

**Table 1:** Meta-analysis of the association between low maternal Hb and infant outcomes

	LBW OR (95% CI)	VLBW OR (95% CI)	PTB OR (95% CI)	SGA OR (95% CI)	Stillbirth OR (95% CI)	Perinatal Mortality OR (95% CI)	Neonatal Mortality OR (95% CI)
**Timing: low maternal Hb (< 110 g/L) by timing**							
Preconception	1.72^b^ (1.31–2.26)	--	1.04 (0.97–1.12)	1.79^b^ (1.39–2.31)	1.23^a^ (0.39–3.86)	--	--
First trimester	1.31 (1.16–1.49)	3.21^a^ (1.06–9.71)	1.22 (1.15–1.31)	1.12 (1.04–1.21)	1.54 (1.12–2.12)	1.61 (0.80–3.24)	1.30^b^ (0.92–1.84)
Second trimester	0.96 (0.72–1.27)	--	1.37 (1.15–1.64)	1.05 (0.94–1.19)	2.22 (1.36–3.65)	1.38^b^ (0.88–2.15)	--
Third trimester	1.45 (1.23–1.70)	2.20^a^ (1.43–3.38)	1.49 (1.30–1.71)	0.89 (0.80–0.98)	1.59 (0.91–2.78)	1.13 (0.73–1.76)	0.90^a^ (0.72–1.12)
**Cutoff: low maternal Hb at any time during pregnancy by cutoff**							
≤ 70 g/L	2.06 (1.63–2.61)	2.85^a^ (2.07–3.92)	2.08 (1.67–2.59)	1.22^b^ (1.09–1.37)	2.85 (1.66–4.90)	4.41^a^ (2.21–8.81)	1.83^b^ (1.52–2.19)
≤ 80 g/L	1.89 (1.61–2.23)	2.85^a^ (2.07–3.92)	1.89 (1.58–2.25)	1.31 (1.14–1.51)	2.27 (1.53–3.38)	3.82 (2.34–6.24)	1.62 (1.37–1.90)
≤ 90 g/L	1.70 (1.51–1.91)	4.95^b^ (1.60-15.29)	1.60 (1.44–1.77)	1.26 (1.17–1.36)	2.01 (1.57–2.56)	2.66 (1.28–5.55)	1.46 (1.30–1.63)
≤ 100 g/L	1.35 (1.26–1.45)	2.87 (1.48–5.57)	1.49 (1.39–1.60)	1.13 (1.04–1.23)	1.73 (1.43–2.10)	2.06 (1.47–2.90)	1.37 (1.26–1.48)
≤ 110 g/L	1.28 (1.22–1.35)	2.15 (1.47–3.13)	1.35 (1.29–1.42)	1.10 (1.02–1.19)	1.43 (1.24–1.65)	1.75 (1.28–2.39)	1.25 (1.16–1.34)
Overall estimate* < 110 g/L	1.28 (1.22–1.35)	2.15 (1.47–3.13)	1.35 (1.29–1.42)	1.10 (1.02–1.19)	1.43 (1.24–1.65)	1.75 (1.28–2.39)	1.25 (1.16–1.34)

^a^ based on 1 study

^b^ based on 2 studies

-- no data available

*Overall estimates using Hb concentrations measured at any point during pregnancy

*Hb* hemoglobin; *LBW* low birthweight; *VLBW* very low birthweight; *PTB* preterm birth; *OR* Odds Ratio; *CI* Confidence Interval

### Low maternal hb and infant outcomes

Overall estimates of low Hb (< 110 g/L) during pregnancy and the relationship to LBW, VLBW, PTB, SGA, stillbirth, perinatal mortality, and neonatal mortality are illustrated in Fig. 2a. Low maternal Hb was significantly associated with odds of LBW (OR (95% CI) 1.28 (1.22–1.35)), VLBW (OR (95% CI) 2.15 (1.47–3.13)), PTB (OR (95% CI) 1.35 (1.29–1.42)), SGA (OR (95% CI) 1.11 (1.02–1.19)), stillbirth (OR (95% CI) 1.43 (1.24–1.65)), perinatal mortality (OR (95% CI) 1.75 (1.28–2.39)), and neonatal mortality (OR (95% CI) 1.25 (1.16–1.34)).

#### By timing

Summary estimates were constructed for low maternal Hb (< 110 g/L) and the odds of LBW, VLBW, PTB, SGA, stillbirth, perinatal mortality, and neonatal mortality by timing at preconception, 1st trimester, 2nd trimester, and 3rd trimester (Table 1). The strongest associations between low maternal Hb and LBW and SGA were observed during the preconception period (OR (95% CI) 1.72 (1.31–2.26)); OR (95% CI) 1.79 (1.39–2.31)), respectively), while relationships were nonsignificant for PTB and stillbirth during this time. During the first trimester, low maternal Hb was associated with increased odds of the following adverse birth outcomes: LBW (OR (95% CI) 1.31 (1.16–1.49)), VLBW (OR (95% CI) 3.21 (1.06–9.71)), PTB (OR (95% CI) 1.22 (1.15–1.31)), SGA (OR (95% CI) 1.12 (1.04–1.21)) and stillbirth (OR (95% CI) 1.54 (1.12–2.12)). During the second trimester, relationships between low maternal Hb and infant outcomes were significant for PTB (OR (95% CI) 1.37 (1.15–1.64)) and stillbirth (OR (95% CI) 2.22 (1.36–3.65)) but not for the other outcomes. During the third trimester, low maternal Hb was associated with increased odds of the following adverse birth outcomes: LBW (OR (95% CI) 1.45 (1.23–1.70)), VLBW (OR (95% CI) 2.20 (1.43–3.38)) and PTB (OR (95% CI) 1.49 (1.30–1.71)) but decreased odds of SGA (OR (95% CI) 0.89 (0.80–0.98)). Data to examine perinatal and neonatal morality by timing were limited; relationships within each time period were non-significant.

#### By cutoff

Summary estimates were created for low Hb and the odds of LBW, VLBW, PTB, SGA, stillbirth, perinatal mortality, and neonatal mortality by Hb concentration cutoff: ≤70, ≤ 80, ≤90, ≤ 100, and ≤ 110 g/L (Table 1). The odds of poor birth outcomes generally increased as Hb concentration decreased across cutoffs, with strongest associations at the lowest cutoff of ≤ 70 g/L for LBW (OR (95% CI) 2.06 (1.63–2.61)), PTB ≤ 70 g/L: (OR (95% CI) 2.08 (1.67–2.59)), stillbirth ≤ 70 g/L: (OR (95% CI) 2.85 (1.66–4.90)), perinatal mortality ≤ 70 g/L (OR (95% CI) 4.41 (2.21–8.81)) and neonatal mortality ≤ 70 g/L: (OR (95% CI) 1.83 (1.52–2.19)). The odds of VLBW were highest at ≤ 90 g/L (OR (95% CI) 4.95 (1.60-15.29)); however, limited data existed at lower cutoffs. Odds of SGA were similar across Hb cutoffs ranging from OR (95% CI) 1.31 (1.14–1.51) for ≤ 80 g/L to OR (95% CI) 1.11 (1.03–1.19) for ≤ 110 g/L.

#### By etiology

Limited information was available to examine the role of etiology of anemia (IDA vs. non-IDA) with regard to birth outcomes (See Supplementary Tables 2, Additional File 1). From the data available, neither IDA (by itself) or non-IDA (by itself) during pregnancy was significantly associated with LBW, SGA, or PTB.

### High maternal hb and infant outcomes

Overall estimates of high Hb (≥ 130 g/L) during pregnancy and relationship to LBW, VLBW, PTB, SGA, stillbirth, perinatal mortality, and neonatal mortality are illustrated in Fig. 2b. High maternal Hb was significantly associated with odds of VLBW (OR (95% CI) 1.35 (1.16–1.57)), PTB (OR (95% CI) 1.12 (1.00-1.25)), SGA (OR (95% CI) 1.17 (1.09–1.25)) and stillbirth (OR (95% CI) 1.32 (1.09–1.60)).

#### By timing

When data were sufficient, summary estimates were constructed for high maternal Hb (≥ 130 g/L) and the odds of LBW, VLBW, PTB, SGA, stillbirth, perinatal mortality, and neonatal mortality by timing at preconception, 1st trimester, 2nd trimester, and 3rd trimester (Table 2). During preconception, maternal high Hb was not associated with any of the infant health outcomes. During the first trimester, high maternal Hb was associated with increased odds of VLBW (OR (95% CI) 1.35 (1.16–1.57)) and stillbirth (OR (95% CI) 1.23 (1.03–1.47)) but not LBW, PTB, SGA, or neonatal mortality. During the second trimester, high maternal Hb was associated with increased odds of LBW (OR (95% CI) 1.40 (1.02–1.93)) and SGA (OR (95% CI) 1.27 (1.08–1.49)) but not PTB, stillbirth or perinatal mortality. During the third trimester, high maternal Hb was associated with increased odds of stillbirth (OR (95% CI) 2.31 (1.30–4.10)) but decreased odds of LBW (OR (95% CI) 0.58 (0.51–0.66)).

#### By cutoff

Summary estimates were created for high Hb and the odds of LBW, VLBW, PTB, SGA, stillbirth, perinatal mortality, and neonatal mortality by Hb concentration cutoff: ≥130, ≥ 140, ≥150, and ≥ 160 g/L (Table 2). Overall, there were limited data to evaluate different cutoffs and patterns were not clear. The exception was stillbirth, for which the odds doubled when shifting from a cutoff of ≥ 130 g/L (OR (95% CI) 1.32 (1.09–1.60)) to a cutoff of ≥ 140 g/L (OR (95% CI) 2.30 (1.38–3.85)).

**Table 2:** Meta-analysis of association between high maternal Hb and infant outcomes

	LBW OR (95% CI)	VLBW OR (95% CI)	PTB OR (95% CI)	SGA OR (95% CI)	Stillbirth OR (95% CI)	Perinatal Mortality OR (95% CI)	Neonatal Mortality OR (95% CI)
**Timing: high maternal Hb (≥ 130 g/L) by timing**							
Preconception	1.04^a^ (0.96–1.12)	--	1.06^b^ (1.00-1.12)	1.02^a^ (0.98–1.07)	0.83^a^ (0.52–1.33)	--	--
First trimester	1.53 (0.69–3.39)	1.35^a^ (1.16–1.57)	1.03 (0.94–1.13)	1.08 (1.00-1.17)	1.23 (1.03–1.47)	---	1.25^a^ (0.63–2.49)
Second trimester	1.40^a^ (1.02–1.93)	--	1.10 (0.96–1.26)	1.27 (1.08–1.49)	1.07^a^ (0.89–1.29)	1.40^a^ (0.86–2.29)	--
Third trimester	0.58^a^ (0.51–0.66)	--	0.85 (0.61–1.18)	1.14^b^ (1.00-1.31)	2.31^a^ (1.30–4.10)	--	--
**Cutoff: high maternal Hb at any time during pregnancy by cutoff**							
≥ 130 g/L	1.50 (0.94–2.39)	1.35^a^ (1.16–1.57)	1.12 (1.00-1.25)	1.17 (1.09–1.25)	1.32 (1.09–1.60)	1.40^a^ (0.86–2.29)	1.21^b^ (0.94–1.57)
≥ 140 g/L	1.37 (0.44–4.23)	1.34^a^ (1.05–1.73)	1.14 (0.91–1.43)	1.16 (0.99–1.35)	2.30 (1.38–3.85)	--	--
≥ 150 g/L	0.78^a^ (0.19–3.18)	1.43^a^ (0.74–2.75)	1.14 (0.77–1.68)	1.11 (0.86–1.44)	--	--	--
≥ 160 g/L	0.78^a^ (0.19–3.18)	--	0.42^a^ (0.14–1.29)	0.61^a^ (0.22–1.67)	--	--	--
Overall estimate* ≥ 130 g/L	1.50 (0.94–2.39)	1.35 (1.16–1.57)	1.12 (1.00-1.25)	1.17 (1.09–1.25)	1.32 (1.09–1.60)	1.40 ^a^ (0.86–2.29)	1.21^b^ (0.94–1.57)

^a^ based on 1 study

^b^ based on 2 studies

-- no data available

*Overall estimates using Hb concentrations measured at any point during pregnancy

*Hb* hemoglobin; *LBW* low birthweight; *VLBW* very low birthweight; *PTB* preterm birth; *SGA* small-for-gestational age

*OR* Odds Ratio; *CI* Confidence Interva

### Maternal outcomes

**Fig. 3:**
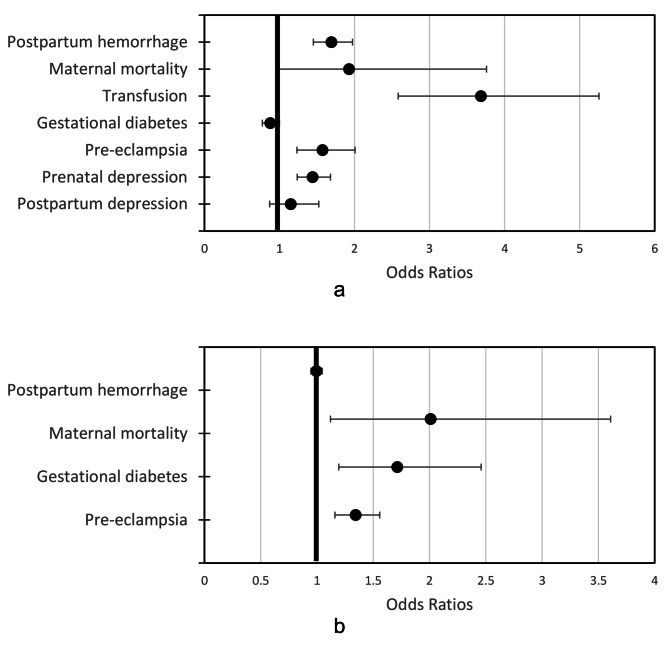
Summary estimates of low (**a**) and high (**b**) hemoglobin concentrations and associations with adverse maternal outcomes

Maternal outcomes with sufficient data for meta-analysis included: (1) post-partum hemorrhage (n = 14); (2) transfusion (n = 10); (3) pre-eclampsia (n = 11); (4) gestational diabetes (n = 7); (5) prenatal depression (n = 3); (6) postpartum depression (n = 3) and (7) maternal mortality (n = 3). Figure 3 provides an overview of associations of maternal Hb and maternal health outcomes; which is further described in Tables 3 and 4 for low and high maternal Hb and stratified by Hb cutoff and timing.

**Table 3:** Meta-analysis of association between high maternal Hb and maternal outcomes

	Post-Partum Hemorrhage OR (95% CI)	Transfusion OR (95% CI)		Pre-Eclampsia OR (95% CI)	Gestational Diabetes OR (95% CI)	Postpartum Depression OR (95% CI)		Prenatal Depression OR (95% CI)		Maternal Mortality OR (95% CI)	
**Timing: low maternal Hb (< 110) by timing**											
Preconception	--	--		--	1.00^a^ (0.97–1.04)	-		--		--	
First trimester	--	--		1.42 (0.69–2.95)	0.92^b^ (0.73–1.16)	--		--		--	
Second trimester	1.43^a^ (1.01–2.03)	1.65^a^ (0.68-4.00)		0.50^a^ (0.29–0.87)	--	1.19^a^ (0.80–1.78)		--		--	
Third trimester	1.58 (1.40–1.79)	6.15 (3.70-10.23)		1.36 (1.00-1.84)	0.86^a^ (0.74-1.00)	1.18 (0.78–1.79)		--		--	
**Cutoff: low maternal Hb at any time during pregnancy by cutoff**											
≤ 70 g/L	3.19^b^ (0.89–11.37)	43.46^a^ (22.06–85.62)		2.83 (2.08–3.85)	--	--		--		3.77^b^ (0.77–18.31)	
≤ 80 g/L	3.19^b^ (0.89–11.70)	24.75^a^ (8.15–75.15)		2.83 (2.08–3.85)	--	--		--		3.77^b^ (0.77–18.31)	
≤ 90 g/L	3.02 (2.04–4.48)	12.07 (6.70-21.72)		2.30 (1.31–4.03)	--	--		--		4.83^b^ (2.17–10.74)	
≤ 100 g/L	1.92 ( 1.42–2.59)	6.45 (3.62–11.49)		1.85 (1.29–2.64)	--	--		1.49^a^ (1.22–1.81)		2.87 (1.08–7.67)	
≤ 110 g/L	1.69 (1.45–1.97)	3.68 (2.58–5.26)		1.57 (1.23–2.01)	0.88 (0.77-1.00)	1.15 (0.87–1.52)		1.44 (1.24–1.68)		1.93 (0.99–3.76)	
Overall estimate* < 110 g/L	1.69 (1.45–1.97)	3.68 (2.58–5.26)		1.57 (1.23–2.01)	0.88 (0.77-1.00)	1.15 (0.87–1.52)		1.44 (1.24–1.68)		1.93 (0.99–3.76)	

^a^ based on 1 study

^b^ based on 2 studies

-- no data available

*Overall estimates using Hb concentrations measured at any point during pregnancy

*Hb* hemoglobin; *OR* Odds Ratio; *CI* Confidence Interval

**Table 4:** Meta-analysis of association between high maternal Hb and maternal outcomes

	Post-Partum Hemorrhage OR (95% CI)	Transfusion OR (95% CI)	Pre-Eclampsia OR (95% CI)	Gestational Diabetes OR (95% CI)	Postpartum Depression OR (95% CI)	Prenatal Depression OR (95% CI)	Maternal Mortality OR (95% CI)
**Timing: high maternal Hb (> 130) by timing**							
Preconception	--	--	--	1.24^a^ (1.11–1.38)	--	--	--
First trimester	--	--	1.61^a^ (0.92–2.84)	1.73^b^ (1.10–2.71)	--	--	--
Second trimester	1.01^a^ (0.95–1.15)	--	1.60^a^ (1.03–2.48)	--	--	--	--
Third trimester	--	--	1.27^a^ (1.18–1.36)	--	--	--	--
**Cutoff: high maternal Hb at any time during pregnancy by cutoff**							
≥ 130 g/L	1.00^b^ (0.95–1.05)	--	1.34 (1.16–1.56)	1.71 (1.19–2.46)	--	--	2.01^a^ (1.12–3.61)
≥ 140 g/L	1.00^b^ (0.87–1.14)	--	1.58^b^ (0.88–2.83)	2.10^b^ (1.65–2.68)	--	--	2.01^a^ (1.12–3.61)
≥ 150 g/L	--	--	2.38^a^ (1.20–4.69)	--	--	--	--
Overall estimate* ≥ 130 g/L	1.00^b^ (0.95–1.05)	--	1.34 (1.16–1.56)	1.71 (1.19–2.46)	--	--	2.01^a^ (1.12–3.61)

^a^ based on 1 study

^b^ based on 2 studies

-- no data available

*Overall estimates using Hb concentrations measured at any point during pregnancy

*Hb* hemoglobin; *OR* Odds Ratio; *CI* Confidence Interval

### Low maternal hb and maternal outcomes

Data across time points and cutoffs were combined to construct summary estimates of the relationship of low Hb (< 110 g/L) to postpartum hemorrhage, maternal mortality, transfusion, gestational diabetes, pre-eclampsia, postnatal depression, and postpartum depression (Fig. 3a). Low maternal Hb was significantly associated with odds of postpartum hemorrhage (OR (95% CI) 1.69 (1.45–1.97)), transfusion (OR (95% CI) 3.68 (2.58–5.26)), pre-eclampsia (OR (95% CI) 1.57 (1.23–2.01)), and prenatal depression (OR (95% CI) 1.44 (1.24–1.68)). We also conducted a sensitivity analysis with revised definition of postpartum blood loss by ≥ 1000 mL and the results remained consistent (OR (95% CI) 1.87 (1.15–3.06)), Figure S2.

#### By timing

Limited data were available to examine the role of timing with regard to low maternal Hb and maternal outcomes. During preconception, only one study reported a (non-significant) association with gestational diabetes and no data were available for other outcomes. During the first trimester, data were only available for pre-eclampsia and gestational diabetes and relationships were nonsignificant. During the second trimester, low maternal Hb increased the odds of post-partum hemorrhage (OR (95% CI) 1.43 (1.01–2.03)) but reduced the odds of pre-eclampsia (OR (95% CI) 0.50 (0.29–0.87)). During the third trimester, low maternal Hb was associated with increased odds of post-partum hemorrhage (OR (95% CI) 1.58 (1.40–1.79)), and transfusion (OR (95% CI) 6.15 (3.70-10.23)).

#### By cutoff

Summary estimates were constructed for low Hb and the odds of postpartum hemorrhage, transfusion, pre-eclampsia, gestational diabetes, postpartum depression, prenatal depression, and maternal mortality by Hb concentration cutoff (Table 3). The odds of postpartum hemorrhage, transfusion and pre-eclampsia increased as Hb concentration decreased with a 1.8 to 11.8-fold increase in ORs when shifting from a cutoff of ≤ 110 g/L to a cutoff of ≤ 70 g/L. For maternal mortality, while the overall results for ≤ 110 g/L were non-significant, at lower cutoffs of ≤ 100 (OR (95% CI) 2.87 (1.08–7.67)), and ≤ 90 (OR (95% CI) 4.83 (2.17–10.74)) relationships were significant.

### High maternal hb and maternal outcomes

Data across time points and cutoffs were combined to construct summary estimates of the relationship of high Hb (≥ 130 g/L) to postpartum hemorrhage, maternal mortality, transfusion, gestational diabetes, pre-eclampsia, prenatal depression, and postpartum depression (Fig. 3b). When all data were combined, high Hb (≥ 130 g/L) was significantly associated with odds of maternal mortality (OR (95% CI) 2.01 (1.12–3.61)), gestational diabetes (OR (95% CI) 1.71 (1.19–2.46)), and pre-eclampsia (OR (95% CI) 1.34 (1.16–1.56)).

#### By timing

Limited data were available to examine associations of high maternal Hb with maternal outcomes. During preconception, high maternal Hb was associated with increased odds of gestational diabetes (OR (95% CI) 1.24 (1.11–1.38)) but data were not available for other outcomes. During the first trimester, high maternal Hb was associated with increased odds of gestational diabetes (OR (95% CI) 1.73 (1.10–2.71)). During the second and third trimester, high maternal Hb was associated with increased odds of pre-eclampsia (OR (95% CI) 1.27 (1.18–1.36)).

#### By cutoff

Summary estimates were created for the relationship of high Hb to post-partum hemorrhage, transfusion, pre-eclampsia, gestational diabetes, postpartum depression, prenatal depression, and maternal mortality by Hb concentration cutoff: ≥130, ≥ 140, and ≥ 150 g/L (Table 4). The odds of post-partum hemorrhage were similar and non-significant for the first two of these Hb cutoffs (≥ 130 g/L: (OR (95% CI) 1.00 (0.95–1.05)); ≥140 g/L (OR (95% CI) 1.00 (0.87–1.14)). The odds of pre-eclampsia were highest when Hb concentration was ≥ 150 g/L (OR (95% CI) 2.38 (1.20–4.69)) compared to a cutoff of ≥ 130 (OR (95% CI) 1.34 (1.16–1.56)). For gestational diabetes, ORs were significant for Hb ≥ 130 g/L (OR (95% CI) 1.71 (1.19–2.46)) and ≥ 140 g/L (OR (95% CI) 2.10 (1.65–2.68)). Data were limited for other outcomes.

## Discussion

Our updated systemic review provides new insights into the critical role of optimal Hb concentrations during preconception and pregnancy. Unique aspects of this review are the dual focus on low and high Hb concentrations and consideration of a range of both maternal and infant health outcomes. During pregnancy, low maternal Hb was associated with increased odds of poor birth outcomes (LBW, VLBW, PTB, SGA, stillbirth, perinatal and neonatal mortality) and adverse maternal outcomes (post-partum hemorrhage, preeclampsia, prenatal depression, blood-transfusion and maternal mortality). Likewise, high maternal Hb was associated with increased odds of poor birth outcomes (VLBW, PTB, SGA, stillbirth) and adverse maternal outcomes (preeclampsia, gestational diabetes and maternal mortality). Reported associations varied by trimester of pregnancy and Hb cutoff values but not anemia etiology.

This review builds on our prior review [15] with the addition of 53 new studies for a total 148 studies and includes new outcomes including maternal depression and maternal mortality in the meta-analyses. This review expands upon prior reviews on maternal anemia and adverse birth outcomes [10, 164–170]. Low maternal Hb during pregnancy, depending on the timing of assessment and cutoff used, was associated with up to nearly a 5-fold increased risk of poor birth outcomes. This review highlights the importance of early prevention and treatment of anemia, before many women even seek antenatal care. The strongest associations with low maternal Hb were noted during the preconception period (for LBW and SGA) and during the first trimester (for VLBW). Throughout pregnancy, low maternal Hb concentrations remained an important predictor of poor birth outcomes. One exception was that low maternal Hb during the third trimester was associated with a reduced risk of SGA. The reason for this association is unclear and could be spurious finding or related to failure of plasma volume expansion [171]. Overall, lower Hb cutoffs were associated with greater risks of adverse outcomes in a dose-response pattern.

The mechanisms underlying the association of low maternal Hb with birth outcomes are complex and multifactorial and may include nutritional deficiencies (e.g., iron, vitamin A, folic acid, or vitamin B12 deficiency), infectious causes (e.g., malaria, schistosomiasis, hookworm infection, HIV), hemoglobinopathies (sickle cell anemia, thalassemia), and inflammation [172]. Iron deficiency has been reported to contribute to up to 75% of all types of anemia during pregnancy [172]. Iron deficiency results from insufficient dietary intake coupled with increased systemic demand, impaired absorption, or blood loss. The prevalence of iron deficiency varies geographically, with higher prevalence in low-income countries. Across pregnancy, there are changes in iron requirements and iron absorption, with decreases in requirements the first trimester followed by a nearly three-fold increase in the third trimester due to increased maternal red blood cell mass expansion, placental demand, and fetal growth [173, 174]. IDA is associated with lower oxygen delivery to the tissues, fatigue, increased risk of infection, and cardiac failure in severe cases [175]. Among offspring, IDA is associated with poor perinatal outcomes including LBW, intrauterine growth restriction, PTB, neonatal anemia. Although iron deficiency has been largely attributed to nutritional causes (e.g., insufficient iron intake or poor iron absorption), several non-nutritional causes may be important to consider as well. Inflammation (due to infectious causes or low-grade inflammation observed in individuals with overweight or obesity) may also impact iron uptake and metabolism via increased hepcidin levels, resulting in anemia of inflammation despite sufficient iron stores [173]. Furthermore, although outside of the scope of the present review, it is important to consider the interplay between hemoglobinopathies and iron deficiency. Recent studies report that thalassemia carriers have altered iron metabolism and erythropoiesis [176–178]. Within our review, relationships between maternal Hb and birth outcomes did not vary by anemia etiology; this is likely attributable to the lack of information across included studies with respect to prevalence of iron deficiency and merits further examination.

Maternal low Hb concentrations were also associated with a range of adverse maternal outcomes. For several outcomes, results were consistent with those of prior reviews [15] (postpartum hemorrhage, transfusion, and pre-eclampsia), but some outcomes were new to this review (prenatal and post-partum depression and maternal mortality). Maternal low Hb during pregnancy was associated with a 44% increased risk of prenatal depression, however, there is insufficient information to understand how this association varies by timing of Hb assessment or cutoff used. Overall, associations between maternal low Hb and maternal mortality were non-significant; however, when lower cutoffs of ≤ 100 g/L and ≤ 90 g/L were used, maternal Hb was associated with nearly a 3 to 5-fold increase in maternal mortality. Data are lacking on the importance of timing of maternal Hb assessment and maternal mortality.

Much of the existing literature has focused on low maternal Hb during pregnancy; however, our review demonstrates that high maternal Hb concentrations during this time are likewise associated with up to a 2-fold increased odds of adverse infant outcomes (VLBW, PTB, SGA and stillbirth) and maternal outcomes (pre-eclampsia, gestational diabetes and maternal mortality). There are several potential mechanisms to consider when reflecting on the maternal health and birth outcomes associated with high Hb concentrations. Plasma volume expansion, which occurs rapidly in the second and third trimester of pregnancy, is a normal physiological process and facilitates the transfer of nutrients to the fetus. High Hb concentrations may be a result of inadequate plasma volume expansion, which has been reported as a risk factor for both SGA and pre-eclampsia [179]. While mechanisms are unclear and there is the potential for reverse causality, there is evidence that abnormal placental vascularization may trigger a pathway of increased production of vasconstrictive agents, reduced plasma volume expansion, and pre-eclampsia [180, 181]. There is increasing attention to the possibility that pre-eclampsia may be a result of a dysfunctional maternal cardiovascular system that is unable to manage plasma volume expansion [182, 183]. High Hb concentrations may also be a result of higher iron status. While higher iron status is generally viewed favorably during pregnancy, iron is a pro-oxidant that can result in DNA damage of placenta cells and interfere with metabolic processes related to glucose metabolism if the body is experiencing iron overload [184–186]. High iron status during pregnancy has been associated with SGA and gestational diabetes in previous studies [34, 187].

Key strengths of this review are the inclusion of both ends of the spectrum for Hb concentrations and the range of adverse outcomes for both the mother and infant. Stratification of results by Hb cutoff and timing of assessment also adds further depth and understanding to these complex relationships. Associations between low Hb and adverse outcomes tend to be stronger earlier in pregnancy while associations between high Hb and adverse outcomes were inconsistent across time points. Mixed findings in the second trimester may point to a need to further examine optimal cutoffs during this period.

Our study is one of the largest systematic reviews to date on this topic, including data collected in 69 different countries over a 30-year time period. As such there is high heterogeneity in our analysis driven by differences in definitions and measurement of exposure (maternal Hb) and outcomes as well as potential true differences by context, perhaps driven by variable etiology of anemia and high Hb. It is important to note that this review examines observational associations that could be confounded by underlying socioeconomic, health or environmental factors and thus cannot establish causality. Furthermore, there are inherent limitations of using dichotomous characterizations of Hb rather than understanding risk across a range of continuous values of Hb [188, 189]. In addition, where data were available we stratified by trimester of pregnancy; however, a closer examination of maternal Hb by week of gestation and application of the new gestational age-specific centiles for maternal hemoglobin concentrations from the INTERGROWTH study would be ideal [16]. Future work with pooled individual level data and standardized adjustment for known confounding factors may allow for greater clarity on associations of hemoglobin level and increased risk of adverse outcomes. Depending on the prevalence of the adverse outcomes, future reporting of relative risk may be preferable [190]. This review is limited by the data available. Notably, there is a lack of data on the etiology of low or high Hb and many gaps remain in understanding the role of timing and cutoffs across preconception and pregnancy. Studies that reported data using multiple Hb cutoffs or time points were more heavily weighted in the overall estimates. Hb is a convenient and widely used biomarker in nutrition and health research, however it remains unclear if the associations with adverse outcomes are being driven by direct alterations in functional Hb for essential functions or by the indirect underlying causes of anemia (e.g. iron deficiency vs. hemoglobinopathy) or high Hb (e.g. excess iron vs. failure of plasma volume expansion). Further research is needed to better understand the implications of the etiology of anemia for redefining optimal Hb cutoffs during pregnancy and optimizing public health programs for women.

## Conclusion

Both low and high hemoglobin concentrations were associated with adverse maternal and infant health outcomes. Our work builds on prior evidence on the importance of maintaining a healthy maternal Hb concentration from preconception to delivery. Further research is needed to define cutoffs for both anemia and high Hb and implement effective interventions to optimize healthy Hb ranges during pregnancy.

## Electronic supplementary material

Below is the link to the electronic supplementary material.

**Additional file 1**

## Data Availability

All data and materials are available upon request (Melissa Young, melissa.young@emory.edu).

## Abbreviations

HB: Hemoglobin
LBW: Low birthweight
PTB: Preterm birth
SGA: Small-for-gestational age
VLBW: Very low birthweight
WHO: World Health Organization
