# Supplementary material for: Maternal low and high hemoglobin concentrations and associations with adverse maternal and infant health outcomes: an updated global systematic review and meta-analysis
Source: BMC Pregnancy Childbirth. 2023 Apr 19;23:264. doi: 10.1186/s12884-023-05489-6 (PMC10114461; doi:10.1186/s12884-023-05489-6)
Supplement: Supplementary file 1 — Additional file 1 [file 12884_2023_5489_MOESM1_ESM.docx]

**ADDITIONAL FILES**

Supplementary Figure S1. Study selection

Supplementary Figure S2: Meta-analysis of association between maternal anemia and post-partum hemorrhage (PPH) as defined by ≥ 1000 mL.

SupplementaryTable S1. Summary of all Observational Studies Included in Meta-Analysis

Supplementary Table S2: Meta-analysis summary of association of IDA and non-IDA with birth outcomes

**Figure S1: Flow of study selection.**

**Total studies reviewed (9874)**

References retrieved for title and abstract review from PubMed and Cochrane (n=9855)

Additional records identified from reference lists (n=19)

Duplicate records removed (n = 57)

Titles and abstracts screened (n = 9798)

Records excluded on basis of inclusion and exclusion criteria (n = 8674)

Assessed for inclusion during full text review (n = 1142)

Excluded: (n=994)

On basis of inclusion and exclusion criteria (n=743)

Unable to contact study authors for pertinent information (n=38)

Unable to translate (n=9)

Full text unavailable (n=26)

Duplication of study results (n=1)

Other statistical measures (n=61)

Other outcomes, insufficient for meta-analysis (n=90)

High risk populations at baseline (n=26)

**Studies included the meta-analysis (n = 148)**

**Infant outcomes:**

low birthweight (n=61); preterm birth (n=63); small for gestational age (n=37); stillbirth (n=27); perinatal mortality (n=13); neonatal mortality (n=10)

**Maternal outcomes:**

Post-partum hemorrhage (n=14); transfusion (n=10); pre-eclampsia (n=11); gestational diabetes (n=7); prenatal depression (n=3); postpartum depression (n=3); maternal mortality (n=3)

PND (n=3); Maternal mortality (n=4)

| **Supplementary Table S1. Summary of all Observational Studies Included in Meta-Analysis** | | | | | | | | |  |
| --- | --- | --- | --- | --- | --- | --- | --- | --- | --- |
| **Study (Author, Year)** | **Setting** | **Study Design** | **Sample Size** | **Exposure** | **Timing of Exposure** | **Maternal and Child Outcomes** | **Covariates** | **Grade** | |
| Abadiga, 2021^1^ | Ethiopia | Case control | 516 | Anemia: Hb <110 g/L | During pregnancy | PTB | Adjusted (factors not provided) | Moderate | |
| Abera, 2019^2^ | Ethiopia | Cross sectional | 380 | Anemia: Hb 11 g/dL | During pregnancy | LBW | Adjusted (factors not provided) | Moderate | |
| Abeysena, 2010^3^ | Sri Lanka | Prospective cohort | 817 | Anemia: Hb < 104 g/L High Hb: > 139 g/L | 1st trimester | LBW, PTB, SGA, GDM | Past history of abortion, past history of LBW, Rate of weight gain (kg/week) | Moderate | |
| Adam, 2013^4^ | Sudan | Case control | 3,290 | Anemia: Hb < 110 g/L | Predelivery | Preeclampsia | Age group in years, parity groups, educational level, prenatal care, placenta previa | High | |
| Adam, 2019^5^ | Ghana | Case control | 360 | Anemia: Hb < 11 g/dL | 1^st^ trimester | LBW | Planned pregnancy, mode of delivery, parity, and previous LBW | High | |
| Adams, 1995^6^ | United States | Retrospective cohort | 1,825 | Anemia: Hb < 110 g/L | First recorded measure | PTB | Medical center | Moderate | |
| Adler, 2019^7^ | Israel | Prospective cohort | 1,346 | Anemia: Hb < 105 g/L | At delivery | PPD | Adjusted (factors not provided) | Moderate | |
| Ahmed, 2018^8^ | Ethiopia | Case control | 286 | Anemia: Hb < 11 g/dL | Hb at booking | LBW | Adjusted (factors not provided) | High | |
| Ajepe, 2020^9^ | Nigeria | Cross sectional | 220 | Anemia: 1st/3rd trimester: <11 g/dL  2nd trimester: <10.5 g/dL | 3^rd^ trimester | LBW, stillbirth, transfusion, neonatal mortality | Adjusted (factors not provided) | Moderate | |
| Alemu, 2020^10^ | Ethiopia | Cross sectional | 290 | Anemia: Hb <110 g/dL | 1st trimester  3rd trimester | LBW | Pregnancy, altitude, socioeconomic status, and ANC attendance | Moderate | |
| Ali, 2011^11^ | Sudan | Case control | 9,578 | Mild/moderate anemia: 70- 109 g/L Severe anemia: < 70 g/L | Predelivery | PTB, LBW, stillbirth | Age, parity, education, residence, and antenatal care | High | |
| Ali, 2020^12^ | Pakistan; India | Prospective cohort | 130,888 | Anemia: Hb <11 g/dL  High Hb: ≥13 g/dL | Hb at booking | LBW, PTB, stillbirth, neonatal mortality | Maternal age, education level, parity, BMI, and gestational age at enrollment | High | |
| Alwan, 2015^13^ | United Kingdom | Retrospective cohort | 362 | Anemia: ≤ 20 weeks: Hb <110 g/L >20 weeks: Hb < 105 g/L | 1st trimester | SGA, PTB | Maternal age, smoking, gestational diabetes, pre-eclampsia, and area deprivation score | High | |
| Ardic, 2019^14^ | Turkey | Retrospective cohort | 483 | Anemia Hb <11 g/dL | Average of 1^st^ and 2^nd^ trimester | PTB | Maternal age, weight gain during pregnancy, level of mother education, baby’s gender, monthly income, and number of pregnancy follow-ups | Moderate | |
| Babu, 2018^15^ | India | Prospective cohort | 823 | Anemia Hb <11 g/dL | During pregnancy | PND | Age, gestational age, and parity |  | |
| Bader, 2010^16^ | Sudan | Case control | 312 | Anemia: Hb <110 g/L | Predelivery | stillbirth | Age, primipara, housewife, education < secondary level, history of miscarriage, history of stillbirth, lack of antenatal care, male neonates, history of malaria, BMI | Moderate | |
| Baig, 2013^17^ | Pakistan | Case control | 600 | Anemia: Hb <100 g/L High Hb: >140 g/L | During pregnancy | PTB | Maternal age, diet pattern | High | |
| Banhidy, 2011^18^ | Hungary | Case control | 60,994 | Anemia: Hb <110 g/L | 1st trimester | PTB, LBW | Maternal age, birth order, and maternal socio-economic status | High | |
| Beckert, 2019^19^ | United States | Retrospective cohort | 2,869,415 | Anemia: 2nd trimester: Hb <10.5 g/dL  3rd trimester: Hb <11 g/dL | During pregnancy | PTB, SGA | Hypertension, diabetes, fibroids, previous preterm birth, previous poor pregnancy outcome, placental abruption, placental insufficiency, placental infarction, chorioamnionitis, maternal blood transfusion, any hysterectomy, unplanned hysterectomy, maternal admission to ICU, unplanned operating room procedure following pregnancy | High | |
| Bian, 2013^20^ | China | Retrospective cohort | 55,633 | Anemia: Hb <100 g/L | 1st trimester | LBW | Neonate gender, maternal age | High | |
| Bilano, 2014^21^ | Multi-country (24) ^1^ | Cross sectional | 276,388 | Severe anemia: Hb <70 g/L | During pregnancy | preeclampsia | Maternal age, marital status, education, BMI, parity, history of chronic hypertension, gestational diabetes, cardiac/ renal disease, pyelonephritis/ urinary tract infection, antenatal care visits, facility capacity, GNI per capita, maternal mortality ratio | High | |
| Bodeau-Livinec, 2011^22^ | Benin | Retrospective cohort | 1,508 | Anemia: Hb <110 g/L Severe anemia: Hb <80 g/L | 2nd trimester 3rd trimester Predelivery | LBW | Malaria at time of Hb assessment, gravidity, BMI, having latrines, having electricity, level of education, and first antenatal care at time of inclusion for Hb during 2nd trimester or number of ANC visits for Hb during the third trimester or at delivery | High | |
| Borah, 2016^23^ | India | Cross sectional | 450 | Anemia: Hb <110 g/L | 3rd trimester | LBW | Age, parity and interpregnancy intervals | Fair | |
| Butwick, 2017^24^ | United States | Case control | 850 | Anemia: Hb ≤99 g/L, Hb 100- 109 g/L | Predelivery | PPH | Maternal age, insurance, race/ethnicity, chronic hypertension, gestational age (wk), number of earlier CDs, type of pregnancy, GDM, placenta previa, previous D&C or D&E, Time of CD, Mode of anesthesia, Uterine incision | High | |
| Chaudhary, 2021^25^ | Nepal | Cross sectional | 4,000 | Anemia: Hb <110 g/L | During pregnancy | SGA | Maternal age, maternal  education, sex of the babies, maternal sleep duration, intake of different foods, solid fuel use for cooking, tobacco use, environmental tobacco smoke, various existing maternal medical conditions | Moderate | |
| Chen, 2017^26^ | China | Retrospective cohort | 1,174 | Anemia: Hb <110 g/L | Early pregnancy Late pregnancy | SGA | Maternal age, educational level, household registration, occupation, number of pregnancy, gestational weeks, infant gender | High | |
| Chen, 2018^27^ | Multi-country (16)^2^ | Retrospective cohort | 214,067 | Severe anemia: Hb <7 g/dL | During pregnancy | Preeclampsia | Age, gravidity, marital status, education, cardiac or renal disease, and country | Moderate | |
| Chu, 2020^28^ | Taiwan | Retrospective cohort | 1,128 | Anemia: Hb <99 g/L, Hb 100-108 g/L | At delivery | LBW, VLBW, PTB, SGA, stillbirth, GDM, PPH | Altitude of residence and smoking | High | |
| Chumak, 2010^29^ | Russia | Retrospective cohort | 24,525 | Anemia: Hb <120 g/L | During pregnancy | stillbirth, PTB, BW | Maternal age, parity, marital status, alcohol consumption, tobacco smoking, and time periods | Moderate | |
| Chumak, 2011^30^ | Russia | Retrospective cohort | 24,526 | Anemia: Hb <90 g/L, 90-99 g/L, 100-109 g/L, 110-119 g/L | During pregnancy | stillbirth, PTB, BW | Maternal age, parity, marital status, alcohol consumption, tobacco smoking, and time periods | Moderate | |
| Cung, 2014^31^ | Palestine | Retrospective cohort | 5,644 | Low Hb: <70 g/L, 70-89 g/L, 90-109 g/L High Hb: ≥145 g/L | Predelivery | stillbirth | Gestational age, birth weight | High | |
| Delpisheh, 2008^32^ | United Kingdom | Case control | 270 | Anemia: Hb <110 g/L | First ANC visit | IUGR, SGA | Gestational age, underweight, and primiparity | Moderate | |
| Dessu, 2020^33^ | Ethiopia | Cross sectional | 1,820 | Low Hb <10 g/dL, 10-11.9 g/dL  High Hb: >12 g/dL | During pregnancy | Perinatal mortality | Number of ANC visits, mode of delivery, gestational age, birth weight, pregnancy induced hypertension | Fair | |
| Domple, 2016^34^ | India | Case control | 320 | Low Hb: <80 g/L | During pregnancy | LBW | Weight gain, gestational age, first ANC visit, education of case, education of husband, bad obstetric history, IFA tablets consumption, planned/unplanned pregnancy or underlying disease | Moderate | |
| Drukker, 2015^35^ | Jerusalem | Retrospective cohort | 75,660 | Mild anemia: Hb 10.0- 10.9 g/dL Moderate/severe anemia: <10.0 g/dL | predelivery | LBW, SGA, LGA, PTB, transfusion, PPH | Maternal age at delivery, maternal education as a proxy for SES and income, nullipara, multipara (2-5), or grand multipara, previous spontaneous abortions, previous c-section, assisted reproductive techniques, neonatal gestational age, induction of labor, epidural analgesia, and birthweight percentile | High | |
| Egbe, 2020^36^ | Cameroon | Case control | 592 | Mild anemia: Hb 90- <110 g/L Moderate anemia: Hb 70- <90 g/L Severe anemia: Hb <70 g/L | Predelivery | Stillbirth | Adjusted (factors not provided) | High | |
| Ehrenthal, 2012^37^ | United States | Retrospective cohort | 59,282 | Anemia: Hb <105 g/L | Predelivery | Transfusion | Demographic, medical, and pregnancy characteristics, gestational age, and birth weight | High | |
| Elhassan, 2010^38^ | Sudan | Case control | 194 | Anemia: Hb <110 g/L | predelivery | LBW | Maternal age, primiparous status, education < secondary, lack of antenatal care, interpregnancy interval, maternal weight, maternal height, maternal BMI | High | |
| Eng, 2016^39^ | Australia | Case control | 164 | High Hb: ≥145 g/L | Hb at booking | stillbirth | Maternal age at delivery, advanced maternal age, higher BMI, Australian by birth, sex of stillbirth, previous stillbirth, previous preterm delivery, previous c-section, primiparity, married, reduced fetal movement in last 2 weeks | Moderate | |
| Ferdous, 2012^40^ | Bangladesh | Prospective cohort | 1,037 | Moderate/severe anemia: Hb <90 g/L | During pregnancy | Perinatal mortality | Age, parity, SES, education | High | |
| Figueiredo, 2019^41^ | Brazil | Prospective cohort | 622 | Anemia: Hb <11 g/dL | During pregnancy | LBW | Maternal age, family income, urinary infection, parity, alcoholic beverage consumption during pregnancy, and gestational BMI | Moderate | |
| Finkelstein, 2020^42^ | Uganda | Prospective cohort | 367 | Anemia:  1st/3rd trimester: Hb <11 g/dL 2nd trimester: Hb <10.5 g/dL | 1st trimester | LBW, PTB, SGA | Antiretroviral treatment arm, gestational age, maternal age, BMI, and log CD4 T-cell counts at enrollment. Hb, hemoglobin; LAZ,  length-for-age z score; WAZ, weight-for-age z score; WLZ, weight-for-length z score | Moderate | |
| Finkelstein, 2020^43^ | India | Prospective cohort | 360 | Anemia:  1st/3rd trimester: Hb <11 g/dL 2nd trimester: Hb <10.5 g/dL | 1^st^ trimester | LBW, PTB, SGA | Gestational age of sample, vitamin B12 intervention, maternal BMI, standard of living index (SLI)>28, educational level, and ln(CRP) | High | |
| Gaillard, 2014^44^ | Netherlands | Prospective cohort | 7,317 | High Hb: ≥132 g/L; Anemia Hb ≤11 | 2nd trimester | LBW, PTB, SGA, preeclampsia | Gestational age at enrollment and at blood sampling, maternal age, BMI, parity, ethnicity, education, alcohol consumption during pregnancy, smoking during pregnancy, folic acid supplement use, and multivitamin use | High | |
| Ganesh Kumar, 2010^45^ | India | Case control | 450 | Anemia: Hb <110 g/L | During pregnancy | LBW | Age, parity, antenatal visits, spacing, weight, height, PIH, h/o abortion, stillbirth | Fair | |
| Geelhoed, 2006^46^ | Ghana | Prospective cohort | 309 | Severe anemia: Hb <80 g/L | lowest Hb during pregnancy, predelivery | LBW, perinatal mortality | Maternal age |  | |
| Getiye, 2017^47^ | Ethiopia | Case control | 1,113 | Anemia: Hb <11 g/dL | During pregnancy | Perinatal mortality | Educational status, birth interval, ANC follow up, TT vaccination, gestational age, mode of delivery, history of ENND, obstetric complications, fetal presentation, newborn weight, congenital anomaly, partograph use | Moderate | |
| Girma, 2019^48^ | Ethiopia | Case control | 279 | Anemia: Hb <110 g/L | During pregnancy | LBW | Adjusted (factors not provided) | Moderate | |
| Gonzales, 2009^49^ | Peru | Retrospective cohort | 35,449 | Anemia: Hb <90, 90-109 g/L High Hb: >129 g/L | First available measurement | PTB, SGA, stillbirth | Maternal age, maternal education, marital status, prior stillbirth or preterm birth, prenatal care, parity, maternal body mass index, placental abruption, and gestational hypertension in current pregnancy | High | |
| Gonzales, 2012^50^ | Peru | Retrospective cohort | 379,816 | Low Hb: <70, 70- <90, 90- <110 g/L High Hb: >145 g/L | 3rd trimester | PPH, preeclampsia | Age, maternal education level, marital status, body mass index, prenatal care, parity, gestational diabetes mellitus and cardiopathy (current pregnancy), gestational age at which Hb was first measured, and migration if delivery was in a place of different altitude to that in which most of the pregnancy occurred | High | |
| Gonzales, 2014^51^ | Peru | Retrospective cohort | 161,909 | Mild anemia: 90-109 g/L Moderate anemia: 70-89 g/L Severe anemia: <70 g/L | 2nd trimester | PTB, stillbirth | Age, BMI, maternal education, antenatal care, parity, preeclampsia, altitude, and urinary infection | High | |
| Goshtasebi, 2013^52^ | Iran | Prospective cohort | 281 | Anemia: Hb <110 g/L | 3rd trimester | PPD | Age, education level, type of delivery, newborn’s sex, gestational age at delivery, Hb at delivery | High | |
| Guignard, 2021^53^ | France | Case control | 4,903 | Mild anemia: 90-109 g/L Moderate anemia: 70-89 g/L Severe anemia: <70 g/L | 3rd trimester | PPH, Preeclampsia | Maternal age (continuous variable); body mass index  (continuous variable); maternal place of birth; single mother; pre-existing medical condition; parity; interpregnancy time  interval < 2 years; multiple pregnancy; non-severe pregnancy-related hypertensive disorders; menorrhagia; macrosomia; and timing of  haemoglobin level measurement | High | |
| Gurung, 2020^54^ | Nepal | Prospective cohort | 63,099 | Anemia: Hb <110 g/L | During pregnancy | PTB | Adjusted (factors not provided) | Fair | |
| Hamaleinen, 2003^55^ | Finland | Case control | 22799 | Anemia: Hb <100 g/L | 1st trimester 2nd trimester 3rd trimester | PTB, LBW, SGA | Adjusted (factors not provided) | High | |
| Hinderaker, 2003^56^ | Tanzania | Prospective cohort | 3,618 | Low Hb: <90, 90-109 g/L High Hb: ≥130 g/L | 2nd trimester | Perinatal mortality | Parity, loss of child | High | |
| Hussein, 2020^57^ | Ghana | Prospective cohort | 1,626 | Anemia: Hb <90 g/L | 3rd trimester | SGA | Maternal malaria, kitchen hours, number of people cooked for, use of disinfectants | Moderate | |
| Hwang, 2010^58^ | South Korea | Retrospective cohort | 3,560 | Anemia: Hb <100 g/L | 3rd trimester | PTB, SGA, perinatal mortality | Adjusted (factors not provided) | Moderate | |
| Jaleel, 2010^59^ | Pakistan | Case control | 124 | Anemia: Hb <90 g/L | During pregnancy | PPH | Age (> 35 years), previous history of PPH, home delivery | Fair | |
| Jessani, 2021^60^ | Democratic Republic of the Congo, Guatemala, India, Kenya, Pakistan, Zambia | Retrospective cohort | 11,976 | Anemia:Hb <110 g/L | 1^st^ trimester | PTB, SGA, Neonatal mortality | Altitude, haemoglobin at  screening, stillbirth, SGA and birthweight <2500 g | High | |
| Kalanda, 2006^61^ | Malawi | Cross sectional | 1,571 | Low Hb: < 80, < 100, < 110 g/L | 2nd trimester | SGA | Malaria parasitemia, gestational age, birth weight | Fair | |
| Kattula, 2014^62^ | India | Prospective cohort | 420 | Anemia: Hb <100 g/L | 2nd trimester | LBW | Offspring gender, preterm birth, < 4 antenatal care visits, beedi work at home | Moderate | |
| Kebede, 2019^63^ | Ethiopia | Cross sectional | 422 | Anemia: Hb <100 g/L | During pregnancy | PPH | Adjusted (factors not provided) | Moderate | |
| Kelkay, 2019^64^ | Ethiopia | Cross sectional | 325 | Anemia: Hb <11 g/dL | During pregnancy | PTB | Adjusted (factors not provided) | Moderate | |
| Khan, 2016^65^ | Pakistan | Case control | 160 | Moderate/ severe anemia: Hb <100 g/L | Predelivery | LBW | Educational level, SES, maternal nutrition, periodontitis | High | |
| Khattar, 2013^66^ | India | Case control | 300 | Severe anemia: Hb <70 g/L | During pregnancy | LBW | preterm pregnancy, lower SES, previous LBW baby, utilization of ANC, ETS exposure | Moderate | |
| Kim, 2020^67^ | Korea | Retrospective cohort | 366,122 | Anemia: Hb <11 g/dL | Preconception | GDM | Maternal age, parity, smoking status, BMI, fasting glucose, BP, TC, AST, and ALT | High | |
| Knottnerus, 1990^68^ | Netherlands | Prospective cohort | 796 | Hb ≥8.0 mmol/l | 3rd trimester | LBW, PTB | Pregnancy-induced hypertension | High | |
| Koura, 2012^69^ | Benin | Prospective cohort | 542 | Anemia: Hb <110 g/L | Predelivery | LBW, PTB | Maternity hospital | Moderate | |
| Kumari, 2019^70^ | India | Cross sectional | 515 | Anemia: Hb <12 g/dL  Mild: Hb 8-9.9 g/dL  Moderate: Hb 10-11.8 g/dL  Severe Hb <8 g/dL | Hb at booking | LBW, PTB | Maternal age at delivery, body mass index, parity, and education level | Moderate | |
| Lake, 2019^71^ | Ethiopia | Cross sectional | 304 | Anemia: Hb <110 g/L | During pregnancy | LBW | Adjusted (factors not provided) | Moderate | |
| Lao, 2002^72^ | China | Prospective cohort | 730 | High Hb >130 g/L | First antenatal visit | GDM | Adjusted (factors not provided) | Moderate | |
| Levy, 2005^73^ | Israel | Retrospective cohort | 153,396 | Anemia: Hb <100 g/L | 1st trimester | PTB, LBW | Ethnicity, maternal age, placental problems, CS, and non-vertex presentation |  | |
| Locks, 2020^74^ | India | Retrospective cohort | 29,253 | Mild anemia: Hb 8-10.9 g/dL  Moderate: Hb 7-7.9 g/dL  Severe: Hb <7 g/dL | 1^st^ trimester | LBW | Maternal age, maternal education, parity, maternal height, gestational age at hemoglobin measure | High | |
| Lone, 2004^75^ | Pakistan | Prospective cohort | 629 | Anemia: Hb <110 g/L | During pregnancy | Stillbirth, LBW, PTB | Adjusted (factors not provided) | High | |
| Maeda, 2020^76^ | Japan | Prospective cohort | 1,128 | Anemia:  1st/3rd trimester: <11 g/dL 2nd trimester: <10.5 g/dL | 2nd trimester  3rd trimester | PPD | Maternal age, parity, maternal education, household income, gestational week at delivery, and mode of delivery | High | |
| Maghsoudlou, 2016^77^ | Iran | Case control | 3,383 | Low Hb: < 110 g/L High Hb: 121- 139, ≥ 140 g/L | Preconception 1st trimester 3rd trimester | stillbirth | Maternal age, pre-pregnancy BMI, maternal height, gestational age at first Hb measurement, parity, smoking status, region of residence, and husband's profession | High | |
| Malhotra, 2002^78^ | India | Prospective cohort | 447 | Low Hb: ≤75, 76-85, 86-95 g/L High Hb: 106-115, ≥ 116 g/L | During pregnancy | LBW | Age, parity | High | |
| Mamun, 2006^79^ | Bangladesh | Prospective cohort | 1584 | Hb: 90-99.9, 100- 109.9, 110- 119.9, ≥ 120 g/L | During pregnancy | Perinatal mortality | Hypertension, antepartum haemorrhage, infection, previous experience of abortions, stillbirth, or newborn death, demographics, socio-economic characteristics, preterm deliveries, maternal age, iron folate supplementation | High | |
| Marchant, 2004^80^ | Tanzania | Cross sectional | 301 | Severe anemia: Hb <80 g/L | During pregnancy | perinatal mortality | Season of recruitment of the pregnant women, gestational month at recruitment, age, parity, education, marital status, MUAC, BMI | Moderate | |
| Marti, 2001^81^ | Venezuela | Case control | 543 | Anemia: <110 g/L | 3rd trimester | PTB | Placental abruption, PROM, previous premature labor, prenatal care visits, and uterine Bleeding during more than one trimester | High | |
| Masukume, 2015^82^ | New Zealand, Australia, England, Ireland | Prospective cohort | 5,609 | Anemia: <110 g/L | 2nd trimester | LBW, PTB, SGA | Country, maternal age, having a marital partner, ethnic origin, years of schooling, and having paid work | High | |
| Meis, 1995^83^ | United Kingdom | Retrospective cohort | 25,844 | Low Hb: <104, 104- 117 g/L High Hb: ≥130 g/L | First antepartum visit | PTB | Age, maternal height, maternal weight, parity, previous abortions, previous stillbirth, maternal smoking, social class, bacteriuria, hypertension-proteinuria, early pregnancy bleeding, late pregnancy bleeding | High | |
| Mekie, 2019^84^ | Ethiopia | Cross sectional | 282 | Anemia: <11 g/dL | During pregnancy | LBW | Adjusted (factors not provided) | Fair | |
| Mekuriyaw, 2020^85^ | Ethiopia | Case control | 405 | Anemia: <11 g/dL | During pregnancy | PTB | Adjusted (factors not provided) | Fair | |
| Mohamed, 2012^86^ | United States | Retrospective cohort | 17,338 | Low Hb: <90, 90- 99, 100-109 g/L High Hb: 120-129, 130-139, ≥ 140 g/L | 3rd trimester | LBW, PTB | Maternal age, body mass index measured during pregnancy and at time of delivery, gravidity, multiple gestation, previous cesarean delivery, thyroid disorder, diabetes mellitus (chronic or gestational), hypertension, street drug use, smoking, alcohol, and infant's sex | High | |
| Mola, 1999^87^ | Papua New Guinea | Retrospective cohort | 21,177 | Low Hb: <80 g/L | 2nd trimester | stillbirth | Syphilis, multiple pregnancy, maternal age > 35 years, highland region of origin, HTN in pregnancy | Moderate | |
| Msuya, 2011^88^ | Tanzania | Prospective cohort | 2,654 | Moderate anemia: Hb 70- 89 g/L Severe anemia: <70 g/L | 3rd trimester | LBW | HIV, malaria, maternal BMI | High | |
| Mumbare, 2012^89^ | India | Case Control | 2,998 | Anemia: Hb <110 g/L | 3rd trimester | LBW | Inadequate ANC, weight <55 kg, height <145 cm, tobacco exposure, HTN, low SES, parity 1, maternal education, paternal education, nuclear family, age of mother | Moderate | |
| Nair, 2017^90^ | United Kingdom | Retrospective cohort | 14,001 | Mild anemia: 100-109 g/L  Moderate anemia: < 100 g/L | First visit  Third trimester | Stillbirth, perinatal mortality | Maternal age, body mass index, parity, smoking status, gestational diabetes, antepartum haemorrhage and pregnancy-induced hypertension during index pregnancy, pre-existing diabetes mellitus, haemoglobinopathies, other medical comorbidities and ethnicity | High | |
| Nsereko, 2020^91^ | Rwanda | Prospective cohort | 421 | Anemia: Hb <11 g/dL | 1st trimester | PTB | Adjusted (factors not provided) | Moderate | |
| Nyflot, 2017^92^ | Norway | Case control | 3,123 | Anemia: Hb ≤ 90 g/L | 1st trimester | PPH | Maternal age, BMI, birth weight | High | |
| Oaks, 2019^93^ | Malawi | Retrospective cohort | 2380 | Anemia: Hb <100 g/L at ≤20 weeks  <100 at 36 weeks | ≤20 weeks; 36 weeks | LBW, PTB, SGA | Gestational age at enrollment, parity, maternal age, education level, household food insecurity, household asset index, α-1-acid glycoprotein at the time the blood sample was drawn, C-reactive protein at the  time the blood sample was drawn, infant sex, maternal BMI at enrollment, maternal malaria at enrollment, and HIV status | High | |
| Obadi, 2018^94^ | Yemen | Case control | 303 | Anemia: Hb <110 g/L | During pregnancy | Stillbirth | Maternal age, mother's education, gestational age, prolonged labor, antenatal care visits, umbilical complications, low birth weight, congenital malformation | Moderate | |
| Ota, 2014^95^ | Multi-country (29) ^4^ | Cross sectional | 245,773 | Severe anemia: Hb <70 g/L | Predelivery | SGA | Maternal age, marital status, education, parity, medical conditions during pregnancy such as chronic hypertension, preeclampsia/eclampsia, malaria/dengue, and HIV/AIDS at the individual level, and capacity of health facilities at the facility level by four categorized HDI groups | High | |
| Parks, 2019^96^ | India; Pakistan | Prospective cohort | 92,247 | Mild anemia: Hb 10-10.9 g/dL  Moderate: Hb 7-9.9 g/dL  Severe Hb <7 g/dL | During pregnancy | LBW, VLBW, PTB, stillbirth, neonatal mortality, maternal mortality | Maternal age, education, parity, cluster, access to hospital delivery. | Moderate | |
| Patel, 2018^97^ | India | Prospective cohort | 72,750 | Mild anemia: Hb 10 - 11 g/dL  Moderate: Hb <10 g/dL | First antenatal visit | Stillbirth, LBW, neonatal mortality | Clustering, mother’s age, education level and parity, along with the three levels of anaemia and three categories of BMI | High | |
| Phaloprakarn, 2008^98^ | Thailand | Retrospective cohort | 874 | High Hb: ≥125 g/L | 1st trimester | PTB, LBW, SGA, GDM, preeclampsia | Preeclampsia, GDM | High | |
| Poespoprodjo, 2008^99^ | Indonesia | Cross sectional | 3,046 | Severe anemia: Hb <70 g/L | Predelivery | LBW | Any parasitemia, Papuan ethnicity, primigravid, prematurity | High | |
| Raisanen, 2014^100^ | Finland | Retrospective cohort | 284,415 | Anemia: Hb <110 g/L | During pregnancy | PTB, SGA, stillbirth, neonatal mortality, preeclampsia, PND | Maternal age, number of prior births in multiparous women, birthweight, fetal sex, IVF, pre-eclampsia, placenta previa, marital status, smoking status and SES | High | |
| Raisanen, 2013^101^ | Finland | Case control | 1,390,742 | Anemia: Hb ≤100 g/L | During pregnancy | PTB* | Maternal age, fetal sex, prior preterm delivery in multiparous women, socio-economic status, smoking, in vitro fertilization, placenta previa, and placental abruption | High | |
| Randall, 2019^102^ | Australia | Retrospective cohort | 31,906 | Anemia: Hb <110 g/L | ≤20 weeks | PTB, SGA, PPH, transfusion | Maternal characteristics (age, BMI, country of birth, parity, SES quintile), pregnancy risk factors (smoking, gestational diabetes  and hypertension, pre-existing diabetes and hypertension, previous uterine surgery, abnormal placenta site, antenatal haemorrhage), and potentially mediating factors (labour onset, mode of birth, perineal tears | High | |
| Randall, 2019^103^ | Australia | Prospective cohort | 38,545 | Anemia: Hb <100 g/L  High Hb: ≥130 | 1st ANC  28 weeks | VLBW, PTB, SGA, PPH, transfusion | Maternal age, parity, BMI, COB, smoking status, quintile of socioeconomic status, spontaneous or assisted conception, gestational diabetes, pre-existing diabetes, gestational hypertension, preexisting hypertension, antepartum haemorrhage, abnormal placentation, previous caesarean section, gestational age birth labour onset, mode of birth, and whether an episiotomy was performed | High | |
| Ray, 2020^104^ | Canada | Retrospective cohort | 737,393 | Low Hb: 125-130 g/L | During pregnancy | Transfusion | Maternal age, parity, world region of origin (Caribbean, East Asia/Pacific, Hispanic America, Middle East/North Africa, South Asia, Sub-Saharan Africa, Western  Nations/Europe versus Canada/long-term resident), residential income quintile (1, 2, 3, 4, unknown versus 5), rural/unknown residence (versus urban) and gestational age | High | |
| Rayis, 2020^105^ | Sudan | Prospective cohort | 259 | Anemia: Hb <108 g/L | During pregnancy | GDM | Maternal education | Moderate | |
| Ren, 2007^106^ | China | Retrospective cohort | 88,149 | Anemia:  Hb <80, 80-99, <110 g/L High Hb: 120-139, 140-159, ≥ 160 g/L | 1st trimester | LBW, PTB, SGA | Maternal age, education, gravidity, and BMI | High | |
| Ribot, 2014^107^ | Spain | Prospective cohort | 282 | Anemia: Hb <110 g/L | 1st trimester 2nd trimester 3rd trimester | PTB | Smoking behavior of mother, maternal age, parity, sex of child, BMI at first visit, SES, iron supplementation per day (mg) | High | |
| Ronkainen, 2019^108^ | Finland | Retrospective cohort | 20,554 | Low Hb: <110  High Hb: >135 | 1st trimester 3rd trimester | PTB, SGA | cohort, maternal smoking and maternal hypertensive disorders,  pre-pregnancy BMI, SES, parity and maternal age at delivery | High | |
| Ronnenberg, 2004^109^ | China | Prospective cohort | 405 | Low Hb: <95, 95-120 g/L | Preconception | LBW, PTB, SGA, FGR^4^ | Maternal age, height and height squared, BMI, education, exposure to dust, noise, and passive smoking, work stress, infant gender, and gestational age, and deficiency of folate, vitamin B12 and B7 | High | |
| Rottenstreich, 2020^110^ | Israel | Case control | 510 | Anemia: Hb <110 g/L | Predelivery | Transfusion | Adjusted (factors not provided) | Moderate | |
| Rukuni, 2016^111^ | United Kingdom | Retrospective cohort | 80,422 | Anemia: Hb <100 g/L | Hb at booking | PPH, transfusion, stillbirth, PTB, LBW, neonatal mortality, preeclampsia | Age, parity, smoking status, ethnicity, socio-economic status, body mass index and chronic kidney disease. | High | |
| Saeed, 2014^112^ | Sudan | Cross sectional | 381 | Moderate/severe anemia: Hb < 90 g/L | Predelivery | LBW | Educational level, type of pregnancy, gestational age, presence of hypertension, renal disease, and bleeding during pregnancy | Moderate | |
| Salunkhe, 2019^113^ | India | Prospective cohort | 1,876 | Anemia: Hb <110 g/L | 1st trimester | LBW | Adjusted (factors not provided) | High | |
| Sari, 2020^114^ | Indonesia | Case control | 386 | Anemia: Hb <110 g/L | During pregnancy | PTB | Maternal education, history of preterm labor | Fair | |
| Scanlon, 2000^115^ | United States | Retrospective cohort | 282,123 | 1st trimester: Hb < 97.5, 97.5- 107, 107-116, 134-143, 143-152, >152 g/L  2nd trimester: Hb <89.5, 89.5-98.5, 98.5-107.5, 116.5-134.5, 134.5-143.5, > 143.5 g/L  3rd trimester: Hb <97.3, 97.3- 106.3, 106.3-115.3, 133.3-142.3, 142.3-151.3, >151.3 g/L | 1st trimester 2nd trimester 3rd trimester | PTB, SGA | Maternal race, age, education level, marital status, pre-pregnancy body mass index, weight gain, and cigarette use during pregnancy | High | |
| Schmiegelow, 2012^116^ | Tanzania | Prospective cohort | 872 | Anemia: Hb <80 g/L | Predelivery | Perinatal mortality | Age, gravidity, education <= primary level, short maternal stature, GA at inclusion >21 weeks, adherence to ANV program, full course of IPTp (>= 2 doses), ever used bed net, pregnancy induced HT, preeclampsia, preeclampsia before ANV4, preterm delivery, SGA, antepartum hemorrhage, placental weight, place of delivery | High | |
| Scholl, 1992^117^ | United States | Prospective cohort | 779 | Anemia: 1st/3rd trimester: Hb <110 g/L 2nd trimester: Hb < 105 g/L | 2nd trimester | LBW, PTB, SGA | Maternal age, parity, ethnicity, prior LBW or preterm delivery, bleeding at entry, gestation at initial blood draw (entry), number of cigarettes smoked per day, and pregnancy BMI | High | |
| Scholl, 1994^118^ | United States | Prospective cohort | 755 | Anemia: Hb <110 g/L | 3rd trimester | LBW | Maternal age, parity, black ethnicity, cigarettes smoked/day, prepregnant body mass index, vaginal bleeding before 28 weeks, dietary energy intake, and protein intake | Moderate | |
| Shankar, 2019^119^ | India | Prospective cohort | 248 | Anemia: 1st/3rd trimester: Hb <110 g/L 2nd trimester: Hb <105 g/L | 1st trimester  2nd trimester  3rd trimester | LBW | Adjusted (factors not provided) | Fair | |
| Sharma, 2015^120^ | Nepal | Case control | 465 | Anemia: Hb <110 g/L | During pregnancy | LBW | History of premature delivery, hard physical work done during pregnancy, current age of mother, height of mother, consuming nutritious food during pregnancy, ethnicity, and family type | High | |
| Shehata, 2017^121^ | Canada | Retrospective cohort | 26,994 | Hb range: 0 - 69 g/L, 70 - 79 g/L, 80 - 89 g/L, 90 -99 g/L, >= 100 g/L | During pregnancy | Transfusion | Discharge year, maternal age admission, gestational age, parity, CVD, infection, obesity, placenta previa, preeclampsia, PPH, ICU visit, antepartum hemorrhage | High | |
| Smith, 2019^122^ | Canada | Retrospective cohort | 523, 669 | Mild anemia: Hb 9-10.9 g/dL  Moderate: Hb 7-8.9 g/dL  Severe Hb <7 g/dL | 3rd trimester | PTB, SGA, stillbirth, perinatal mortality, neonatal mortality, preeclampsia, PPH, transfusion | Models adjusted for maternal age, parity, prepregnancy weight, smoking, previous cesarean delivery, alcohol use, and preexisting hypertension | High | |
| Smithers, 2014^123^ | Australia | Retrospective cohort | 96,290 | Anemia: Hb <110 g/L | 1st trimester | LBW, PTB^5^ | Singleton/twin, maternal age, smoking in pregnancy, number of antenatal visits, parity, inter-pregnancy interval, maternal occupation, paternal occupation, maternal Aboriginal or Torres Strait Islander status, the Index of Relative Socio-economic Disadvantage and for living in a remote/not remote area | High | |
| Soysal, 2019^124^ | Turkey | Retrospective cohort | 332 | Anemia: Hb <110 g/L | Predelivery | PTB | Biological age, pregnancy follow-up status, smoking, drug use, history of abortion | High | |
| Steer, 1995^125^ | United Kingdom | Retrospective cohort | 153,602 | Anemia: Hb ≤85, 86-95 g/L High Hb: 106- 115, 116-125, 126- 135, 136-145, > 145 g/L | lowest Hb during pregnancy | LBW, PTB | Ethnic group, BMI, parity, maternal age, and smoking | High | |
| Stephansson, 2000^126^ | Sweden | Case control | 1,404 | Low Hb: ≤ 115, 116-125 g/L High Hb: 136-145, ≥ 146 g/L | 1st trimester | Stillbirth | Maternal age, height, occupation, smoking, BMI, and week of 1st Hb concentration measurement | High | |
| Sun, 2019^127^ | Taiwan | Retrospective cohort | 1,536,796 | Anemia: 1st/3rd trimester: Hb <11 g/dL 2nd trimester: Hb <10.5 g/dL | During pregnancy | Stillbirth | Adjusted (factors not provided) | Fair | |
| Sun, 2021^128^ | China | Retrospective cohort | 46,578 | Anemia: 1st/3rd trimester: Hb <11 g/dL 2nd trimester: Hb <10.5 g/dL | 1st trimester  2nd trimester 3rd trimester | Stillbirth, PTB, SGA | Maternal age, pre-pregnancy body mass index, parity (nulliparous, parous), education (college  education or not), mother's residential status (residents or immigrants), and neonatal sex | High | |
| Symington, 2019^129^ | South Africa | Prospective cohort | 250 | Anemia: 1st/3rd trimester: Hb <11 g/dL 2nd trimester: Hb <10.5 g/dL | <18 weeks; 22 weeks; 36 weeks | LBW, PTB | Maternal age, gestational age at birth, sex of the baby, parity, living standards measure, HIV status, maternal BMI at enrollment, and glucose tolerance | High | |
| Tandu-Umba, 2015^130^ | Congo | Cross sectional | 412 | Anemia: Hb <100 g/L | Predelivery | PTB, SGA, stillbirth | Age < 18 yrs, age ≥ 35 years, single status, previous miscarriage, grand multiparity, diabetes in family, previous prematurity, previous LBW, overweight/obesity, previous cesarean section and previous pre-eclampsia | High | |
| Thakur, 2013^131^ | India | Retrospective cohort | 283 | Anemia: Hb <100 g/L | 1st trimester | neonatal mortality | Socioeconomic status,malnutrition,antenatal care (adequate/ inadequate), pregnancy induced hypertension,antepartum haemorrhage | Moderate | |
| Tsu, 1993^132^ | Zimbabwe | Case control | 450 | Hb: Hb <120 g/L | 3rd trimester | PPH | Age, parity, obstetric history, antenatal hospitalization, and facility booked | High | |
| Tzur, 2012^133^ | Israel | Retrospective cohort | 33,888 | Anemia: Hb <100 g/L | 1st trimester | PPH, transfusion, IUGR, PTB, SGA, LBW, perinatal mortality | Maternal age, ethnicity, previous PTD, cervical incompetence, hypertensive disorders, IUGR, and labor induction | High | |
| Unger, 2015^134^ | Papua New Guinea | Prospective cohort | 671 | Anemia: Hb <90 g/L | During pregnancy | SGA | Gravidity, gestational age at fetal weight measurement | Moderate | |
| Verhoeff, 2001^135^ | Malawi | Prospective cohort | 1,423 | Anemia: Hb <80 g/L | At booking  At delivery | SGA | Primipara, less than 4 ANC visits, height < 150 cm, MUAC < 23 cm, malaria at delivery | High | |
| Walker, 2003^136^ | Jamaica | Retrospective cohort | 234 | Low Hb: <95 g/L | 2nd trimester | LBW | Gestational age, first antenatal visit after 20 weeks, height of mother, weight gain of mother after 20 weeks, consumption of alcohol during pregnancy, young maternal age, hypertension, weight in late pregnancy | Moderate | |
| Wang, 2018^137^ | China | Retrospective cohort | 21,577 | Hb <110 g/L, 130 g/L ≤ Hb < 150 g/L; Hb ≥ 150 g/L | 1^st^ trimester | Preeclampsia, PTB, GDM | Maternal age, pre-pregnancy BMI, gravidity (< 2, ≥ 2), parity (yes, no), education level (≤ 12, > 12), and gestational age at the time of Hb measurement | High | |
| Woldetensay, 2018^138^ | Ethiopia | Prospective cohort | 4,680 | Anemia: Hb <110 g/L | During pregnancy | PND | Adjusted (factors not provided) | Moderate | |
| Xiong, 2003^139^ | China | Retrospective cohort | 14,510 | Anemia: Hb <100 g/L | 1st trimester 3rd trimester | perinatal mortality, PTB, SGA, IUGR, LBW | Hospital stay, maternal age, maternal education, parity, gestational age at the first prenatal visit, BMI at the first visit, hypertensive disorders in pregnancy, vaginal bleeding and prior spontaneous abortion | High | |
| Yatich, 2010^140^ | Ghana | Cross sectional | 746 | Moderate anemia: Hb <110 g/L Severe anemia: Hb <80 g/L | Predelivery | stillbirth | Age (per 5 years), single, low serum folate, no SP doses, past induced abortion, past stillbirth, malaria, infection | Moderate | |
| Yi, 2013^141^ | Korea | Retrospective cohort | 70,895 | Anemia: Hb <120, <100, 100-119, ≥150 g/L | Preconception | PTB, LBW, SGA | Maternal age at delivery, prepregnancy body mass index, parity, education and the result of a health-screening examination | High | |
| Yuniati, 2020^142^ | Indonesia | Prospective cohort | 294 | Anemia: Hb <11 g/dL | 1st trimester | LBW, SGA | Maternal age, pre-pregnancy BMI, and parity | Moderate | |
| Zenebe, 2020^143^ | Ethiopia | Retrospective cohort | 529 | Anemia: Hb <11 g/dL | During pregnancy | LBW | Adjusted (factors not provided) | Moderate | |
| Zhang, 1992^144^ | China | Case control | 865 | Anemia: Hb <105 g/L | During pregnancy | stillbirth | Sex, parity, maternal age | High | |
| Zhang, 1993^145^ | China | Case control | 865 | Anemia: Hb < 80, 80-100 g/L | During pregnancy | neonatal mortality | Sex, gravidity, maternal age, threatened abortion, PIH | Moderate | |
| Zhang, 2018^146^ | China | Prospective cohort | 2,722,274 | Hb range: < 70, 70 - 99, 100- 109, 150-159, 160-169, ≥ 170 g/L | Preconception | PTB | Characteristics of women (age, education, ethnic, occupation, region with GDP per capita), smoking, passive smoking and alcohol drinking status at baseline, history of diseases (diabetes, hypertension and thyroid dysfunction), pre-pregnancy BMI, parity, history of adverse pregnancy outcomes and sex of the child | High | |
| Zhang, 2018^147^ | China | Retrospective cohort | 10,430 | Hb ≤ 119 g/L, 120 - 129 g/L, ≥140 g/L | 1st trimester  2nd trimester | PTB | Education, occupation, gestational age at the first visit, BMI, maternal age, and treatment group | High | |
| Zhou, 1998^148^ | China | Prospective cohort | 829 | Hb: < 90, 90-99, 100-109, < 110, 120-129, ≥ 130 g/L | 1st trimester | LBW, PTB, SGA | Maternal age, gravidity, parity, height, weight, BMI, blood pressure, and infant sex | Moderate | |

GDM, Gestational Diabetes; HB, Hemoglobin; IUGR, Intrauterine growth restriction; LBW, Low birthweight; PPD, Postpartum depression; PPH, Postpartum Hemorrhage; PND, Prenatal Depression; PTB, Preterm birth; SGA, Small-for-gestational age; VLBW, Very low birthweight

**OSM Table S2. Meta-analysis summary of association of IDA and non-IDA with birth outcomes**

| **Outcomes** | **IDA OR (95% CI)** | **Non-IDA OR (95% CI)** |
| --- | --- | --- |
| LBW | 1.54 (0.71-3.36) | 1.10^c^(0.70-1.71) |
| SGA | 1.04^c^ (0.91-1.19) | 1.16^b^ (0.60-2.23) |
| PTB | 1.71 (0.93-3.13) | 1.32^c^ (0.73-2.39) |

IDA defined as hemoglobin <110g/L and serum ferritin < 100 μg/L ^b^ based on 2 studies
^c^ based on 3 studies

**Figure S2: Meta-analysis of association between maternal anemia and post-partum hemorrhage (PPH) as defined** **by ≥ 1000 mL.**

**References**

1. Abadiga M, Wakuma B, Oluma A, Fekadu G, Hiko N, Mosisa G. Determinants of preterm birth among women delivered in public hospitals of Western Ethiopia, 2020: Unmatched case-control study. PLoS One. 2021;16(1):e0245825.

2. Abera Z, Ejara D, Gebremedhin S. Nutritional and non-nutritional factors associated with low birth weight in Sawula Town, Gamo Gofa Zone, Southern Ethiopia. BMC Res Notes. 2019; 12(1):540.

3. Abeysena C, Jayawardana P, de ASR. Maternal haemoglobin level at booking visit and its effect on adverse pregnancy outcome. Aust N Z J Obstet Gynaecol. 2010;50(5):423-7.

4. Adam I, Haggaz AD, Mirghani OA, Elhassan EM. Placenta previa and pre-eclampsia: analyses of 1645 cases at medani maternity hospital, Sudan. Front Physiol. 2013;4:32.

5. Adam Z, Ameme DK, Nortey P, Afari EA, Kenu E. Determinants of low birth weight in neonates born in three hospitals in Brong Ahafo region, Ghana, 2016- an unmatched case-control study. BMC Pregnancy Childbirth. 2019;19(1):174.

6. Adams MM, Sarno AP, Harlass FE, Rawlings JS, Read JA. Risk factors for preterm delivery in a healthy cohort. Epidemiology. 1995;6(5):525-32.

7. Adler L, Tsamir J, Katz R, Koren G, Yehoshua I. Associations of sociodemographic and clinical factors with perinatal depression among Israeli women: a cross-sectional study. BMC Psychiatry. 2019;19(1):331.

8. Ahmed S, Hassen K, Wakayo T. A health facility based case-control study on determinants of low birth weight in Dassie town, Northeast Ethiopia: the role of nutritional factors. Nutr J. 2018;17(1):103.

9. Ajepe AA, Okunade KS, Sekumade AI, Daramola ES, Beke MO, Ijasan O, Olowoselu OF, Afolabi BB. Prevalence and foetomaternal effects of iron deficiency anaemia among pregnant women in Lagos, Nigeria. PLoS One. 2020;15(1):e0227965.

10. Alemu B, Gashu D. Association of maternal anthropometry, hemoglobin and serum zinc concentration during pregnancy with birth weight. Early Hum Dev. 2020;142:104949.

11. Ali AA, Rayis DA, Abdallah TM, Elbashir MI, Adam I. Severe anaemia is associated with a higher risk for preeclampsia and poor perinatal outcomes in Kassala hospital, eastern Sudan. BMC Res Notes. 2011;4:311.

12. Ali SA, Tikmani SS, Saleem S, Patel AB, Hibberd PL, Goudar SS, Dhaded S, Derman RJ, Moore JL, McClure EM, Goldenber RL. Hemoglobin concentrations and adverse birth outcomes in South Asian pregnant women: findings from a prospective Maternal and Neonatal Health Registry. Reprod Health. 2020;17(Suppl 2):154.

13. Alwan NA, Cade JE, McArdle HJ, Greenwood DC, Hayes HE, Simpson NA. Maternal iron status in early pregnancy and birth outcomes: insights from the Baby's Vascular health and Iron in Pregnancy study. Br J Nutr 2015;113(12):1985-92.

14. Ardic C, Usta O, Omar E, Yildiz C, Memis E, Zeren Ozturk G. Relationship between anaemia during pregnancy and preterm delivery. J Obstet Gynaecol 2019;39(7):903-6.

15. Babu GR, Murthy GVS, Singh N, et al. Sociodemographic and Medical Risk Factors Associated With Antepartum Depression. Front Public Health 2018;6:127.

16. Bader E, Alhaj AM, Hussan AA, Adam I. Malaria and stillbirth in Omdurman Maternity Hospital, Sudan. Int J Gynaecol Obstet. 2010;109(2):144-6.

17. Baig SA, Khan N, Baqai T, Fatima A, Karim SA, Aziz S. Preterm birth and its associated risk factors. A study at tertiary care hospitals of Karachi, Pakistan. J Pak Med Assoc. 2013;63(3):414-8.

18. Banhidy F, Acs N, Puho EH, Czeizel AE. Iron deficiency anemia: pregnancy outcomes with or without iron supplementation. Nutrition. 2011;27(1):65-72.

19. Beckert RH, Baer RJ, Anderson JG, Jelliffe-Pawlowski LL, Rogers EE. Maternal anemia and pregnancy outcomes: a population-based study. J Perinatol. 2019;39(7):911-9.

20. Bian Y, Zhang Z, Liu Q, Wu D, Wang S. Maternal risk factors for low birth weight for term births in a developed region in China: a hospital-based study of 55,633 pregnancies. J Biomed Res. 2013;27(1):14-22.

21. Bilano VL, Ota E, Ganchimeg T, Mori R, Souza JP. Risk factors of pre-eclampsia/eclampsia and its adverse outcomes in low- and middle-income countries: a WHO secondary analysis. PLoS One. 2014;9(3):e91198.

22. Bodeau-Livinec F, Briand V, Berger J, Xiong X, Massougbodji A, Day KP, Cot M. Maternal anemia in Benin: prevalence, risk factors, and association with low birth weight. Am J Trop Med Hyg. 2011;85(3):414-20.

23. Borah M, Agarwalla R. Maternal and socio-demographic determinants of low birth weight (LBW): A community-based study in a rural block of Assam. J Postgrad Med. 2016;62(3): 178-81.

24. Butwick AJ, Ramachandran B, Hegde P, Riley ET, El-Sayed YY, Nelson LM. Risk Factors for Severe Postpartum Hemorrhage After Cesarean Delivery: Case-Control Studies. Anesth Analg. 2017;125(2):523-32.

25. Chaudhary N, Yadav SN, Kalra SK, Pathak S, Gupta BK Shrestha S, Patel M, Satia I, Sadhra S, Bolton CE, Kurmi OM. Prognostic factors associated with small for gestational age babies in a tertiary care hospital of Western Nepal: A cross-sectional study. Health Sci Rep. 2021;4(1):e250.

26. Chen C, Grewal J, Betran AP, Vogel JP, Souza JP, Zhang J. Severe anemia, sickle cell disease, and thalassemia as risk factors for hypertensive disorders in pregnancy in developing countries. Pregnancy Hypertension. 2018;13:141-7.

27. Chen JH, Guo XF, Liu S, Long JH, Zhang GQ, Huang MC, Qiu XQ. [Impact and changes of maternal hemoglobin on birth weight in pregnant women of Zhuang Nationality, in Guangxi]. Zhonghua Liu Xing Bing Xue Za Zhi. 2017;38(2):154-7.

28. Chu FC, Shaw SW, Lo LM, Hsieh TT, Hung TH. Association between maternal anemia at admission for delivery and adverse perinatal outcomes. J Chin Med Assoc. 2020;83(4):402-7.

29. Chumak EL, Grjibovski AM. Anemia in pregnancy and its association with pregnancy outcomes in the Arctic Russian town of Monchegorsk, 1973-2002. Int J Circumpolar Health. 2010;69(3):265-77.

30. Chumak EL, Grjibovski AM. Association between different levels of hemoglobin in pregnancy and pregnancy outcomes: a registry-based study in Northwest Russia. Int J Circumpolar Health. 2011;70(5):457-9.

31. Cung TG, Paus AS, Aghbar A, Kiserud T, Hinderaker SG. Stillbirths at a hospital in Nablus, 2010: a cohort study. Glob Health Action. 2014;7:25222.

32. Delpisheh A, Brabin L, Drummond S, Brabin BJ. Prenatal smoking exposure and asymmetric fetal growth restriction. Ann Hum Biol. 2008;35(6):573-83.

33. Dessu S, Dawit Z. Perinatal Mortality and Associated Factors Among Antenatal Care Attended Pregnant Mothers at Public Hospitals in Gamo Zone, Southern Ethiopia. Front Pediatr. 2020;8:586747.

34. Domple VK, Doibale MK, Nair A, Rajput PS. Assessment of maternal risk factors associated with low birth weight neonates at a tertiary hospital, Nanded, Maharashtra. Niger Med J. 2016;57(1):37-43.

35. Drukker L, Hants Y, Farkash R, Ruchlemer R, Samueloff A, Grisaru-Granovsky S. Iron deficiency anemia at admission for labor and delivery is associated with an increased risk for Cesarean section and adverse maternal and neonatal outcomes. Transfusion. 2015;55(12):2799-806.

36. Egbe TO, Ewane EN, Tendongfor N. Stillbirth rates and associated risk factors at the Buea and Limbe regional hospitals, Cameroon: a case-control study. BMC Pregnancy Childbirth. 2020;20(1):75.

37. Ehrenthal DB, Chichester ML, Cole OS, Jiang X. Maternal risk factors for peripartum transfusion. J Womens Health (Larchmt). 2012;21(7):792-7.

38. Elhassan EM, Abbaker AO, Haggaz AD, Abubaker MS, Adam I. Anaemia and low birth weight in Medani, Hospital Sudan. BMC Res Notes. 2010;3:181.

39. Eng C, Karki S, Trivedi AN. Risk factors of stillbirths in Victoria (Australia): A case-control study. J Obstet Gynaecol. 2016;36(6):754-7.

40. Ferdous J, Ahmed A, Dasgupta SK, Jahan M, Huda FA, Ronsmans C, Koblinsky M, Chowdhury ME. Occurrence and determinants of postpartum maternal morbidities and disabilities among women in Matlab, Bangladesh. J Health Popul Nutr. 2012;30(2):143-58.

41. Figueiredo ACMG, Gomes-Filho IS, Batista JET, Orrico GS, Porto ECL, Pimenta RMC, Conceição SDS, Brito SM, Ramos, MdSX, Sena MCF, et al. Maternal anemia and birth weight: A prospective cohort study. PLoS One. 2019;14(3):e0212817.

42. Finkelstein JL, Herman HS, Plenty A, Mehta S, Natureeba P, Clark TD, Kamya MR, Ruel T, Charlebois ED, Cohan D, et al. Anemia and Micronutrient status during pregnancy, and their associations with obstetric and infant outcomes among HIV-Infected Ugandan women receiving antiretroviral therapy. Curr Dev Nutr. 2020;4(5):nzaa075.

43. Finkelstein JL, Kurpad AV, Bose B, Thomas T, Srinivasan K, Duggan C. Anaemia and iron deficiency in pregnancy and adverse perinatal outcomes in Southern India. Eur J Clin Nutr. 2020;74(1):112-25.

44. Gaillard R, Eilers PH, Yassine S, Hofman A, Steegers EA, Jaddoe VW. Risk factors and consequences of maternal anaemia and elevated haemoglobin levels during pregnancy: a population-based prospective cohort study. Paediatr Perinat Epidemiol. 2014;28(3):213-26.

45. Ganesh Kumar S, Harsha Kumar HN, Jayaram S, Kotian MS. Determinants of low birth weight: a case control study in a district hospital in Karnataka. Indian J Pediatr. 2010;77(1):87-9.

46. Geelhoed D, Agadzi F, Visser L, Ablordeppey E, Asare K, O'Rourke P, Leeuwen JSV, Roosmalen JV. Maternal and fetal outcome after severe anemia in pregnancy in rural Ghana. Acta Obstet Gynecol Scand. 2006;85(1):49-55.

47. Getiye Y, Fantahun M. Factors associated with perinatal mortality among public health deliveries in Addis Ababa, Ethiopia, an unmatched case control study. BMC Pregnancy Childbirth. 2017;17(1):245.

48. Girma S, Fikadu T, Agdew E, Haftu D, Gedamu G, Dewana Z, Getachew B. Factors associated with low birthweight among newborns delivered at public health facilities of Nekemte town, West Ethiopia: a case control study. BMC Pregnancy Childbirth. 2019;19(1):220.

49. Gonzales GF, Steenland K, Tapia V. Maternal hemoglobin level and fetal outcome at low and high altitudes. Am J Physiol Regul Integr Comp Physiol. 2009;297(5):R1477-85.

50. Gonzales GF, Tapia V, Gasco M. Correcting haemoglobin cut-offs to define anaemia in high-altitude pregnant women in Peru reduces adverse perinatal outcomes. Arch Gynecol Obstet. 2014;290(1):65-74.

51. Gonzales GF, Tapia V, Gasco M, Carrillo CE, Fort AL. Association of hemoglobin values at booking with adverse maternal outcomes among Peruvian populations living at different altitudes. Int J Gynaecol Obstet. 2012;117(2):134-9.

52. Goshtasebi A, Alizadeh M, Gandevani SB. Association between maternal anaemia and postpartum depression in an urban sample of pregnant women in Iran. J Health Popul Nutr. 2013;31(3):398-402.

53. Guignard J, Deneux-Tharaux C, Seco A, [Beucher](https://pubmed.ncbi.nlm.nih.gov/?term=Beucher+G&cauthor_id=32845522) G, [Kayem](https://pubmed.ncbi.nlm.nih.gov/?term=Kayem+G&cauthor_id=32845522) G, [Bonnet](https://pubmed.ncbi.nlm.nih.gov/?term=Bonnet+MP&cauthor_id=32845522) M-P, [EPIMOMS group](https://pubmed.ncbi.nlm.nih.gov/?term=EPIMOMS+group%5BCorporate+Author%5D). Gestational anaemia and severe acute maternal morbidity: a population-based study. Anaesthesia. 2021;76(1):61-71.

54. Gurung A, Wrammert J, Sunny AK, Gurung R, Rana N, Basaula YN, Paudel P, Pokhrel A, Kc Al. Incidence, risk factors and consequences of preterm birth - findings from a multi-centric observational study for 14 months in Nepal. Arch Public Health. 2020;78:64.

55. Hamalainen H, Hakkarainen K, Heinonen S. Anaemia in the first but not in the second or third trimester is a risk factor for low birth weight. Clin Nut.r 2003;22(3):271-5.

56. Hinderaker SG, Olsen BE, Bergsjo PB, Gasheka P, Lie RT, Kvale G. Perinatal mortality in northern rural Tanzania. J Health Popul Nutr. 2003;21(1):8-17.

57. Hussein H, Shamsipour M, Yunesian M, Hasanvand MS, Fotouhi A. Association of adverse birth outcomes with exposure to fuel type use: A prospective cohort study in the northern region of Ghana. Heliyon. 2020; 6(6):e04169.

58. Hwang HS, Kim YH, Kwon JY, Park YW. Uterine and umbilical artery Doppler velocimetry as a predictor for adverse pregnancy outcomes in pregnant women with anemia. J Perinat Med. 2010;38(5):467-71.

59. Jaleel R, Khan A. Post-partum haemorrhage--a risk factor analysis. Mymensingh Med J. 2010;19(2):282-9.

60. Jessani S, Saleem S, Hoffman MK, Goudar SS, Derman RJ, Moore JL, Garces A, Figueroa L, Krebs NF, Okitawutshu J, et al. Association of haemoglobin levels in the first trimester and at 26-30 weeks with fetal and neonatal outcomes: a secondary analysis of the Global Network for Women's and Children's Health's ASPIRIN Trial. BJOG. 2021;128(9):1487-96.

61. Kalanda BF, Verhoeff FH, Chimsuku L, Harper G, Brabin BJ. Adverse birth outcomes in a malarious area. Epidemiol Infect. 2006;134(3):659-66.

62. Kattula D, Sarkar R, Sivarathinaswamy P, Vasanthakumar V, Venugopal S, Naumova EN, Muliyil J, Ward H, Kang G. The first 1000 days of life: prenatal and postnatal risk factors for morbidity and growth in a birth cohort in southern India. BMJ Open. 2014;4(7):e005404.

63. Kebede BA, Abdo RA, Anshebo AA, Gebremariam BM. Prevalence and predictors of primary postpartum hemorrhage: An implication for designing effective intervention at selected hospitals, Southern Ethiopia. PLoS One. 2019;14(10):e0224579.

64. Kelkay B, Omer A, Teferi Y, Moges Y. Factors Associated with Singleton Preterm Birth in Shire Suhul General Hospital, Northern Ethiopia, 2018. J Pregnancy. 2019;2019:4629101.

65. Khan NS, Ashraf RN, Noor S, Mahmood-ur-Rahman, Mashhadi SF, Rashid Z, Sajjad F, Nazar AF, Nazar HS, Syed R. Association of maternal periodontitis with low birth weight in newborns in a tertiary care hospital. J Ayub Med Coll Abbottabad. 2016;28(1):120-5.

66. Khattar D, Awasthi S, Das V. Residential environmental tobacco smoke exposure during pregnancy and low birth weight of neonates: case control study in a public hospital in Lucknow, India. Indian Pediatr. 2013;50(1):134-8.

67. Kim HY, Kim J, Noh E, Ahn KH, Cho GJ, Hong S-C, Oh M-J, Kim HJ. Prepregnancy hemoglobin levels and gestational diabetes mellitus in pregnancy. Diabetes Res Clin Pract. 2021;171:108608.

68. Knottnerus JA, Delgado LR, Knipschild PG, Essed GG, Smits F. Haematologic parameters and pregnancy outcome. A prospective cohort study in the third trimester. J Clin Epidemiol. 1990;43(5):461-6.

69. Koura GK, Ouedraogo S, Le Port A, Waatier L, Cottrell G, Guerra J, Choudat I, Rachas A, Bouscaillou J, Massougbodji A, Garcia A. Anaemia during pregnancy: impact on birth outcome and infant haemoglobin level during the first 18 months of life. Trop Med Int Health. 2012;17(3):283-91.

70. Kumari S, Garg N, Kumar A, Guru PKI, Ansari S, Anwar S, Singh KP, Kumari P, Mishra PK, Gupta BK, et al. Maternal and severe anaemia in delivering women is associated with risk of preterm and low birth weight: A cross sectional study from Jharkhand, India. One Health. 2019 8:100098.

71. Lake EA, Olana Fite R. Low birth weight and its associated factors among newborns delivered at Wolaita Sodo University Teaching and Referral Hospital, Southern Ethiopia, 2018. Int J Pediatr. 2019;2019:4628301.

72. Lao TT, Chan LY, Tam KF, Ho LF. Maternal hemoglobin and risk of gestational diabetes mellitus in Chinese women. Obstet Gynecol. 2002;99(5 Pt 1):807-12.

73. Levy A, Fraser D, Katz M, Mazor M, Sheiner E. Maternal anemia during pregnancy is an independent risk factor for low birthweight and preterm delivery. Eur J Obstet Gynecol Reprod Biol. 2005;122(2):82-6.

74. Locks LM, Patel A, Katz E, Simmons E, Hibberd P. Seasonal trends and maternal characteristics as predictors of maternal undernutrition and low birthweight in Eastern Maharashtra, India. Matern Child Nutr. 2021;17(2):e13087.

75. Lone FW, Qureshi RN, Emanuel F. Maternal anaemia and its impact on perinatal outcome. Trop Med Int Health. 2004;9(4):486-90.

76. Maeda Y, Ogawa K, Morisaki N, Tachibana Y, Horikawa R, Sago H. Association between perinatal anemia and postpartum depression: A prospective cohort study of Japanese women. Int J Gynaecol Obstet. 2020;148(1):48-52.

77. Maghsoudlou S, Cnattingius S, Stephansson O, Arabi M, Semnani S, Montgomery SM, Bahmanya S. Maternal haemoglobin concentrations before and during pregnancy and stillbirth risk: a population-based case-control study. BMC Pregnancy Childbirth. 2016;16(1):135.

78. Malhotra M, Sharma JB, Batra S, Sharma S, Murthy NS, Arora R. Maternal and perinatal outcome in varying degrees of anemia. Int J Gynaecol Obstet. 2002;79(2):93-100.

79. Mamun AA, Padmadas SS, Khatun M. Maternal health during pregnancy and perinatal mortality in Bangladesh: evidence from a large-scale community-based clinical trial. Paediatr Perinat Epidemiol. 2006;20(6):482-90.

80. Marchant T, Schellenberg JA, Nathan R, Abdulla S, Mukasa O, Mshinda H, Lengeler C. Anaemia in pregnancy and infant mortality in Tanzania. Trop Med Int Health. 20049(2):262-6.

81. Marti A, Pena-Marti G, Munoz S, Lanas F, Comunian G. Association between prematurity and maternal anemia in Venezuelan pregnant women during third trimester at labor. Arch Latinoam Nutr. 2001;51(1):44-8.

82. Masukume G, Khashan AS, Kenny LC, Baker PN, Nelson G, Consortium S. Risk factors and birth outcomes of anaemia in early pregnancy in a nulliparous cohort. PLoS One. 2015;10(4):e0122729.

83. Meis PJ, Michielutte R, Peters TJ, Wells HB, Sands RE, Coles EC, Johns KA. Factors associated with preterm birth in Cardiff, Wales. I. Univariable and multivariable analysis. Am J Obstet Gynecol. 1995;173(2):590-6.

84. Mekie M, Taklual W. Magnitude of low birth weight and maternal risk factors among women who delivered in Debre Tabor Hospital, Amhara Region, Ethiopia: a facility based cross-sectional study. Ital J Pediatr. 2019;45(1):86.

85. Mekuriyaw AM, Mihret MS, Yismaw AE. Determinants of Preterm Birth among Women Who Gave Birth in Amhara Region Referral Hospitals, Northern Ethiopia, 2018: Institutional Based Case Control Study. Int J Pediatr. 2020;2020:1854073.

86. Mohamed MA, Ahmad T, Macri C, Aly H. Racial disparities in maternal hemoglobin concentrations and pregnancy outcomes. J Perinat Med. 2012;40(2):141-9.

87. Mola G, Permezel M, Amoa AB, Klufio CA. Anaemia and perinatal outcome in Port Moresby. Aust N Z J Obstet Gynaecol. 1999;39(1):31-4.

88. Msuya SE, Hussein TH, Uriyo J, Sam NE, Stray-Pedersen B. Anaemia among pregnant women in northern Tanzania: prevalence, risk factors and effect on perinatal outcomes. Tanzan J Health Res. 2011;13(1):33-9.

89. Mumbare SS, Maindarkar G, Darade R, Yenge S, Tolani MK, Patole K. Maternal risk factors associated with term low birth weight neonates: a matched-pair case control study. Indian Pediatr. 2012;49(1):25-8.

90. Nair M, Churchill D, Robinson S, Nelson-Piercy C, Stanworth SJ, Knight M. Association between maternal haemoglobin and stillbirth: a cohort study among a multi-ethnic population in England. British journal of haematology. 2017;179(5):829-37.

91. Nsereko E, Uwase A, Mukabutera A, Muvunyi CM, Rulisa S, Ntirushwas D, Moreland P, Corwin EJ, Santos N, Nzayirambaho M, Wojcicki JM. Maternal genitourinary infections and poor nutritional status increase risk of preterm birth in Gasabo District, Rwanda: a prospective, longitudinal, cohort study. BMC pregnancy and childbirth. 2020;20(1):345.

92. Nyflot LT, Sandven I, Stray-Pedersen B, Pettersen S, Al-Zirqi I, Rosenberg M, Jacobsen AF, Vangen S. Risk factors for severe postpartum hemorrhage: a case-control study. BMC pregnancy and childbirth. 2017;17(1):17.

93. Oaks BM, Jorgensen JM, Baldiviez LM, Adu-afarwuah S, Maleta K, Okronipa H, Sadalaki J, Lartey A, Ashorn P, et al. Prenatal Iron Deficiency and Replete Iron Status Are Associated with Adverse Birth Outcomes, but Associations Differ in Ghana and Malawi. J Nutr. 2019;149(3):513-21.

94. Obadi MA, Taher R, Qayad M, Khader YS. Risk factors of stillbirth in Yemen. J Neonatal Perinatal Med. 2018;11(2):131-6.

95. Ota E, Ganchimeg T, Morisaki N, Vogel JP, Pileggi C, Ortiz-Panozo, Souza JP, Mori R, WHO Multi-Country Survey on Maternal and Newborn Health Research Network. Risk factors and adverse perinatal outcomes among term and preterm infants born small-for-gestational-age: secondary analyses of the WHO Multi-Country Survey on Maternal and Newborn Health. PLoS One. 2014;9(8):e105155.

96. Parks S, Hoffman MK, Goudar SS, Patel A, Saleem S, Ali SA, Goldenberg RL, Hibberd PL, Moore J, Wallace, et al. Maternal anaemia and maternal, fetal, and neonatal outcomes in a prospective cohort study in India and Pakistan. BJOG. 2019;**126**(6):737-43.

97. Patel A, Prakash AA, Das PK, Gupta S, Pusdekar YV, Hibberd PL. Maternal anemia and underweight as determinants of pregnancy outcomes: cohort study in eastern rural Maharashtra, India. BMJ Open. 2018;8(8):e021623.

98. Phaloprakarn C, Tangjitgamol S. Impact of high maternal hemoglobin at first antenatal visit on pregnancy outcomes: a cohort study. J Perinat Med. 2008;36(2):115-9.

99. Poespoprodjo JR, Fobia W, Kenangalem E, Lampah DA, Warikar N, Seal A, McGready R, Sugiarto P, Tjitra E, Anstey NM, Price RN. Adverse pregnancy outcomes in an area where multidrug-resistant plasmodium vivax and Plasmodium falciparum infections are endemic. Clin Infect Dis. 2008;46(9):1374-81.

100. Raisanen S, Gissler M, Saari J, Kramer M, Heinonen S. Contribution of risk factors to extremely, very and moderately preterm births - register-based analysis of 1,390,742 singleton births. PLoS One. 2013;8(4):e60660.

101. Raisanen S, Kancherla V, Gissler M, Kramer MR, Heinonen S. Adverse perinatal outcomes associated with moderate or severe maternal anaemia based on parity in Finland during 2006-10. Paediatr Perinat Epidemiol. 2014;28(5):372-80.

102. Randall DA, Patterson JA, Gallimore F, Morris JM, McGee TM, Ford JB, Obstetric Transfusion Steering Group. The association between haemoglobin levels in the first 20 weeks of pregnancy and pregnancy outcomes. PLoS One. 2019;14(11):e0225123.

103. Randall DA, Patterson JA, Gallimore F, Morris JM, Simpson JM, McGee TM, Ford JB, Obstetric Transfusion Steering Group. Haemoglobin trajectories during pregnancy and associated outcomes using pooled maternity and hospitalization data from two tertiary hospitals. Vox Sang. 2019;114(8):842-52.

104. Ray JG, Davidson A, Berger H, Dayan N, Park AL. Haemoglobin levels in early pregnancy and severe maternal morbidity: population-based cohort study. BJOG. 2020;127(9):1154-64.

105. Rayis DA, Musa IR, Al-Shafei AI, Moheldein AH, El-Gendy OA, Adam I. High haemoglobin levels in early pregnancy and gestational diabetes mellitus among Sudanese women. J Obstet Gynaecol. 2021;41(3):385-9.

106. Ren A, Wang J, Ye RW, Li S, Liu JM, Li Z. Low first-trimester hemoglobin and low birth weight, preterm birth and small for gestational age newborns. Int J Gynaecol Obstet. 2007;98(2):124-8.

107. Ribot B, Isern R, Hernandez-Martinez C, Canals J, Aranda N, Arija V. [Effects of tobacco habit, second-hand smoking and smoking cessation during pregnancy on newborn's health]. Med Clin (Barc). 2014;143(2):57-63.

108. Ronkainen J, Lowry E, Heiskala A, Uusitalo L, Koivunen P, Kajantie E, Vääräsmäki M, Järvelin M-R, Sebert S. Maternal hemoglobin associates with preterm delivery and small for gestational age in two Finnish birth cohorts. Eur J Obstet Gynecol Reprod Biol. 2019;238:44-8.

109. Ronnenberg AG, Wood RJ, Wang X, Xing H, Chen C, Chen D, Guang W, Huang A, Wang L, Xu X. Preconception hemoglobin and ferritin concentrations are associated with pregnancy outcome in a prospective cohort of Chinese women. J Nutr. 2004;134(10):2586-91.

110. Rottenstreich A, Regev N, Levin G, Ezra Y, Yagel S, Sompolinsky Y, Mankuta D, Kalish Y, Elchalal U. Factors associated with postcesarean blood transfusion: a case control study. J Matern Fetal Neonatal Med. 2022;35(3):495-502.

111. Rukuni R, Bhattacharya S, Murphy MF, Roberts D, Stanworth SJ, Knight M. Maternal and neonatal outcomes of antenatal anemia in a Scottish population: a retrospective cohort study. Acta Obstet Gynecol Scand. 2016;95(5):555-64.

112. Saeed OA, Ahmed HA, Ibrahim AM, Mahmood EA, Abdu-Allah TO. Risk factors of low birth weight at three hospitals in Khartoum State, Sudan. Sudan J Paediatr. 2014;14(2):22-8.

113. Salunkhe AH, Pratinidhi AK, Salunkhe JA, Kakade SV, Mohite VR, Patange RP. Antenatal Risk Scoring Scale for Predication of Low Birth Weight and Its Validity. Indian J Community Med. 2019;44(2):97-101.

114. Sari IM, Adisasmita AC, Prasetyo S, Amelia D, Purnamasari R. Effect of premature rupture of membranes on preterm labor: a case-control study in Cilegon, Indonesia. Epidemiol Health. 2020;42:e2020025.

115. Scanlon KS, Yip R, Schieve LA, Cogswell ME. High and low hemoglobin levels during pregnancy: differential risks for preterm birth and small for gestational age. Obstet Gynecol. 2000;96(5 Pt 1):741-8.

116. Schmiegelow C, Minja D, Oesterholt M, Pehrspm C. Suhrs HE, Boström S, Lemnge M, Magistrado P, Rasch V Lusingu J, et al. Factors associated with and causes of perinatal mortality in northeastern Tanzania. Acta Obstet Gynecol Scand. 2012;91(9):1061-8.

117. Scholl TO, Hediger ML, Fischer RL, Shearer JW. Anemia vs iron deficiency: increased risk of preterm delivery in a prospective study. Am J Clin Nutr. 1992;55(5):985-8.

118. Scholl TO, Hediger ML. Anemia and iron-deficiency anemia: compilation of data on pregnancy outcome. Am J Clin Nutr. 1994;59(2 Suppl):492S-500S discussion S-1S.

119. Shankar H, Kumar N, Sandhir R, Sing MP, Mittal S, Adhikari T, Tarique M, Kaur P, Radhika MS, Kumar A, Rao DN. Association of dietary intake below recommendations and micronutrient deficiencies during pregnancy and low birthweight. J Perinat Med. 2019;47(7):724-31.

120. Sharma SR, Giri S, Timalsina U, Bhandari SS, Basyal B, Wagle K, Shrestha L. Low birth weight at term and its determinants in a tertiary hospital of Nepal: a case-control study. PLoS One. 2015;10(4):e0123962.

121. Shehata N, Chasse M, Colas JA, Murphy M, Forster AJ, Malinowski AK, Ducharme R, Fergusson DA, Tinmouth A, Wilson K. Risks and trends of red blood cell transfusion in obstetric patients: a retrospective study of 45,213 deliveries using administrative data. Transfusion. 2017;57(9):2197-205.

122. Smith C, Teng F, Branch E, Chu S, Joseph KS. Maternal and perinatal morbidity and mortality associated with anemia in pregnancy. Obstet Gynecol. 2019;134(6):1234-44.

123. Smithers LG, Gialamas A, Scheil W, Brinkman S, Lynch JW. Anaemia of pregnancy, perinatal outcomes and children's developmental vulnerability: a whole-of-population study. Paediatr Perinat Epidemiol. 2014;28(5):381-90.

124. Soysal S, Sarioz A, Anik Ilhan G, Kocagoz A, Dizi A, Gursoy I, Celik I, Ozmen D. Evaluation of late adolescent pregnancies: Is late adolescence a risk factor for preterm labor? J Matern Fetal Neonatal Med. 2019;32(5):851-6.

125. Steer P, Alam MA, Wadsworth J, Welch A. Relation between maternal haemoglobin concentration and birth weight in different ethnic groups. Bmj 1995; 310(6978): 489-91.

126. Stephansson O, Dickman PW, Johansson A, Cnattingius S. Maternal hemoglobin concentration during pregnancy and risk of stillbirth. Jama 2000; 284(20): 2611-7.

127. Sun CC, Chou HH, Chuang LL. Trends and risk factors of stillbirth in Taiwan 2006-2013: a population-based study. Arch Gynecol Obstet. 2019;299(4):961-7.

128. Sun C-F, Liu H, Hao Y-H, Hu H-T, Zhou -H, Zou K-X, Liu X-M, Sheng J-, Ding G-L, Huang H-F. Association between gestational anemia in different trimesters and neonatal outcomes: a retrospective longitudinal cohort study. World J Pediatr. 2021;17(2):197-204.

129. Symington EA, Baumgartner J, Malan L,Wise AJ. Ricco C. Zandberg L, Smuts CM. Maternal iron-deficiency is associated with premature birth and higher birth weight despite routine antenatal iron supplementation in an urban South African setting: The NuPED prospective study. PLoS One. 2019;14(9):e0221299.

130. Tandu-Umba B, Mbangama AM. Association of maternal anemia with other risk factors in occurrence of Great obstetrical syndromes at university clinics, Kinshasa, DR Congo. BMC Pregnancy Childbirth. 2015;15:183.

131. Thakur N, Saili A, Kumar A, Kumar V. Predictors of mortality of extremely low birthweight babies in a tertiary care centre of a developing country. Postgrad Med J. 2013;89(1058):679-84.

132. Tsu VD. Postpartum haemorrhage in Zimbabwe: a risk factor analysis. Br J Obstet Gynaecol. 1993;100(4):327-33.

133. Tzur T, Weintraub AY, Sergienko R, Sheiner E. Can anemia in the first trimester predict obstetrical complications later in pregnancy? J Matern Fetal Neonatal Med. 2012;25(11):2454-7.

134. Unger HW, Ome-Kaius M, Karl S, Singirok D, Siba P, Walker J, Wangnapi RA, Mueller I, Rogerson SJ. Factors associated with ultrasound-aided detection of suboptimal fetal growth in a malaria-endemic area in Papua New Guinea. BMC Pregnancy Childbirth. 2015;15: 83.

135. Verhoeff FH, Brabin BJ, van Buuren S, Chimsuku L, Kazembe P, Wit JM, Broadhead RL. An analysis of intra-uterine growth retardation in rural Malawi. Eur J Clin Nutr. 2001;55(8):682-9.

136. Walker SP, Ewan-Whyte C, Chang SM, Powell CA, Fletcher H, McDonald D, Grantham-McGregor. Factors associated with size and proportionality at birth in term Jamaican infants. J Health Popul Nutr. 2003;21(2):117-26.

137. Wang C, Lin L, Su R, Zhu W, Wei Y, Yan J, Feng H, Li B, Li S, Yang H. Hemoglobin levels during the first trimester of pregnancy are associated with the risk of gestational diabetes mellitus, pre-eclampsia and preterm birth in Chinese women: a retrospective study. BMC Pregnancy Childbirth. 2018;18(1):263.

138. Woldetensay YK, Belachew T, Biesalski HK, Ghosh S, Lacruz ME, Scherbaum V, Kantelhardt. The role of nutrition, intimate partner violence and social support in prenatal depressive symptoms in rural Ethiopia: community based birth cohort study. BMC Pregnancy Childbirth. 2018;18(1):374.

139. Xiong X, Buekens P, Fraser WD, Guo Z. Anemia during pregnancy in a Chinese population. Int J Gynaecol Obstet. 2003;83(2):159-64.

140. Yatich NJ, Funkhouser E, Ehiri JE, Agbenyega T, Stiles JK, Rayner JC, Turpin A, Ellis WO, Jiang Y, Williams JH, et al. Malaria, intestinal helminths and other risk factors for stillbirth in Ghana. Infect Dis Obstet Gynecol. 2010;2010:350763.

141. Yi SW, Han YJ, Ohrr H. Anemia before pregnancy and risk of preterm birth, low birth weight and small-for-gestational-age birth in Korean women. Eur J Clin Nutr. 2013;67(4):337-42.

142. Yuniati T, Judistiani RTD, Natalia YA, Irianti S, Madjid TH, Ghozali M, Sribudiani Y, Indrati AR, Abdula R, Setiabudiawan B. First trimester maternal vitamin D, ferritin, hemoglobin level and their associations with neonatal birthweight: Result from cohort study on vitamin D status and its impact during pregnancy and childhood in Indonesia. J Neonatal Perinatal Med. 2020;13(1):63-9.

143. Zenebe A, Eshetu B, Gebremedhin S. Association between maternal HIV infection and birthweight in a tertiary hospital in southern Ethiopia: retrospective cohort study. Ital J Pediatr. 2020;46(1):70.

144. Zhang J, Cai WW. Risk factors associated with antepartum fetal death. Early Hum Dev. 1992;28(3):193-200.

145. Zhang J, Cai WW, Lee DJ. Pregnancy-induced hypertension and early neonatal death: a case-control study. Am J Perinatol. 1993;10(5):401-3.

146. Zhang X, Xu Q, Yang Y, Wang L, Liu F, Li Q, Ji M, He Y, Wang Y, Zhang Y, et al. Preconception Hb concentration and risk of preterm birth in over 2.7 million Chinese women aged 20-49 years: a population-based cohort study. Br J Nutr. 2018;120(5):508-16.

147. Zhang Y, Li Z, Li H, et al. Maternal haemoglobin concentration and risk of preterm birth in a Chinese population. J Obstet Gynaecol. 2018;38(1): 32-7.

148. Zhou LM, Yang WW, Hua JZ, Deng CQ, Tao X, Stoltzfus RJ. Relation of hemoglobin measured at different times in pregnancy to preterm birth and low birth weight in Shanghai, China. Am J Epidemiol. 1998;148(10):998-1006.
